# PLOD2 promotes proliferation, migration and invasion of colorectal cancer cells via PI3K-AKT-GSK3β signaling pathway

**DOI:** 10.1038/s41598-026-38593-6

**Published:** 2026-02-10

**Authors:** Hua Fang, Jing Zheng, Shutong Ren, Danjing Chen, Yunli Wu, Xian-E Peng

**Affiliations:** 1 Department of Epidemiology and Health Statistics, Fujian Provincial Key Laboratory of Environment Factors and Cancer, School of Public Health, Fujian Medical University, Fuzhou, China; 2 Key Laboratory of Gastrointestinal Cancer (Fujian Medical University), Ministry of Education, Fuzhou, China

**Keywords:** PLOD2, Colorectal cancer, Malignant progression, Metastasis, PI3K-AKT- GSK3β, Cancer, Cell biology, Oncology

## Abstract

Colorectal cancer (CRC) progression critically depends on the tumor microenvironment. PLOD2, an enzyme involved in collagen biosynthesis, is highly expressed in many cancers. While it promotes CRC growth via the USP15–AKT/mTOR pathway, its role in enhancing tumor cell migration and invasion remains unclear. Our study identified a significant upregulation of PLOD2 in colorectal cancer. This upregulation was closely associated with clinical stage, lymph node metastasis, and nerve invasion in CRC. Functional assays, including CCK-8, colony formation, wound healing, and Transwell migration and invasion assays, showed that PLOD2 overexpression enhanced CRC cell proliferation, migration, and invasion, while PLOD2 silencing exerted the opposite effects. Kyoto Encyclopedia of Genes and Genomes pathway analysis suggested that PLOD2 may influence CRC progression via the PI3K-AKT signaling pathway. Co-immunoprecipitation assays demonstrated that PLOD2 was co-precipitated with PI3K, confirming their interaction. Additionally, rescue experiments showed that the PI3K inhibitor LY294002 and the agonist 740Y-P could reverse PLOD2-mediated effects on CRC cell proliferation, migration, and invasion. This study demonstrates that PLOD2 promotes the proliferation, migration, and invasion of CRC cells by interacting with PI3K to activate the PI3K-AKT-GSK3β signaling pathway.

**Supplementary Information:**

The online version contains supplementary material available at 10.1038/s41598-026-38593-6.

## Introduction

Colorectal cancer (CRC) is one of the most common malignant tumors worldwide^1^. Due to low early detection rates, the majority of patients present are diagnosed advanced-stage disease at diagnosis, resulting in unfavorable prognosis and diminished survival outcomes^2^. Therefore, it is crucial to identify effective biomarkers for the early detection and prognosis of CRC. Tumor progression is co-determined by tumor cells and their microenvironment. The extracellular matrix (ECM) serves as a crucial component of tumor development. As the most abundant protein and primary constituent of the ECM, collagen exhibits dual roles in both normal and cancerous tissue homeostasis^3,4^. Collagen generates biochemical and biophysical signals through its interactions with tumor cells. These signals regulate cell migration, invasion, and proliferation, thereby contributing to tumor progression^5–7^. This study revealed that procollagen-lysine, 2-oxoglutarate 5-dioxygenase 2 (PLOD2), a key enzyme mediating collagen synthesis and cross-linking, was significantly upregulated in CRC tissues.

Recent studies have further emphasized the clinical significance of identifying reliable biomarkers for CRC progression. Inflammation- and nutrition-based biomarkers have been shown to predict disease outcomes and reflect tumor–host interactions^8,9^, supporting the rationale for exploring PLOD2 as a potential prognostic and mechanistic biomarker. Additionally, emerging evidence from single-cell transcriptomic analyses highlights the importance of microenvironmental immune regulation—such as IL27RA-mediated signaling^10^—in shaping CRC behavior, further underscoring the relevance of investigating extracellular matrix–related regulators like PLOD2.

PLOD2 is a member of the PLOD protein family that catalyzes lysine residue hydroxylation for the formation of stable collagen cross-links^11^. This enzyme mediates pyridinoline cross-linking reactions, thereby enhancing collagen stability^12,13^. Hypoxia and TGF-β1 have been shown to induce PLOD2 overexpression in cervical cancer tissues. This upregulation promotes the migration, invasion, and adhesion of cervical cancer cells^14,15^. PLOD2 also promotes cancer cell proliferation, migration, and invasion through activation of the PI3K-AKT signaling pathway in glioma and non-small cell lung cancer^16,17^, Additionally, PLOD2 plays a crucial role in the malignant progression of breast, esophageal, and gastric cancers^18–20^. However, the regulatory mechanism of PLOD2 needs to be further explored.

PI3Ks are key intracellular kinases that regulate cell proliferation, apoptosis, and differentiation. The PI3K–AKT pathway is essential for many physiological processes and is especially important in tumor development. This pathway regulates cell survival, metastasis, and metabolism, and plays a role in angiogenesis and the recruitment of inflammatory factors. The PI3K-AKT signaling pathway plays a crucial role in the progression of various cancers, and studies have demonstrated that PLOD2 contributes to cancer progression through this pathway. For instance, in endometrial cancer cells under hypoxic conditions, PLOD2 expression is elevated, promoting migration, invasion, and epithelial-mesenchymal transition via the PI3K-AKT signaling pathway. Similarly, in glioma, PLOD2 inhibition inactivates the PI3K–AKT pathway and regulates downstream epithelial–mesenchymal transition (EMT)-related factors. Additionally, HIF-1α can induce PLOD2 expression under hypoxia, further promoting hypoxia-induced EMT in glioma cells^16^. The PI3K-AKT pathway has been implicated in the development of several cancers, including breast, gastric, and prostate cancers^21–23^. In our study, we found that PLOD2-related genes were enriched in the PI3K-Akt signaling pathway. Although previous studies have reported that PLOD2 promotes colorectal cancer progression through the USP15–AKT/mTOR axis^24^, whether PLOD2 directly modulates upstream signaling components remains unclear. Notably, no study to date has demonstrated a physical interaction between PLOD2 and PI3K, a key initiator of the PI3K–AKT pathway. In this study, we aimed to fill this gap by investigating whether PLOD2 directly interacts with PI3K and thereby activated downstream PI3K–AKT–GSK3β signaling to promote CRC progression.

## Methods

### Bioinformatics

Differentially expressed genes (DEGs) between CRC and adjacent normal tissues were identified using the GSE97689 dataset from the Gene Expression Omnibus (GEO) database (https://www.ncbi.nlm.nih.gov/geo/). Data analysis was performed using the limma package (version: 3.64.0), with DEGs defined as those showing absolute log2(fold change) > 0 and adjusted *P*-value < 0.05 in volcano plot analysis. Gene Ontology (GO) and Kyoto Encyclopedia of Genes and Genomes (KEGG) enrichment analyses^25^ were conducted using the clusterProfiler R package (version: 4.16.0). *P*-values were adjusted for multiple testing using the Benjamini–Hochberg (BH) method. Enriched pathways with adjusted *P*-value < 0.05 were considered significant. The top 10 most significantly enriched pathways were selected for visualization. The University of Alabama at Birmingham Cancer Data Analysis Portal (UALCAN, http://ualcan.path.uab.edu/) online analysis tool^26^ was used to analyze differences of PLOD2 protein expression in colon cancer based on the Clinical Proteomic Tumor Analysis Consortium (CPTAC, https://cptac-data-portal.georgetown.edu/). Using the Kaplan-Meier plotter (https://kmplot.com/analysis/) online analysis tool and data from The Cancer Genome Atlas (TCGA, https://portal.gdc.cancer.gov), European Genome-Phenome Archive (EGA, https://ega-archive.org/), and GEO databases, we assessed the relationship between PLOD2 expression and overall patient survival in colorectal cancer patients. Using the ‘Similar Gene Detection’ module in GEPIA2, we obtained the top 500 PLOD2-related genes from the TCGA database across all cancer and normal tissues. These datasets were combined for KEGG pathway enrichment analysis.

### Study participants and tissue specimens

The study included patients with newly diagnosed colorectal cancer from April 2014 to April 2016 at Fujian Medical University Union Hospital. All diagnoses were confirmed by postoperative pathological examination, and none of the patients had undergone radiotherapy, chemotherapy, or other anti-tumor adjuvant treatments before surgery. The inclusion criteria were: (1) patients had no contraindications to surgery; (2) no patient received neoadjuvant radiotherapy or chemotherapy before surgery; (3) all surgically resected specimens were clearly typed by pathological diagnosis and confirmed by two pathologists; (4) all cases had complete clinical data. The exclusion criteria were: (1) cases that were not first visit; (2) Concurrent malignant tumours in other parts of the body or immunodeficiency diseases. The study was approved by the Ethical Review Committee of Fujian Medical University Union Hospital (Approval No. 20130501), and all enrolled patients provided written consent for participation.

### Immunohistochemistry (IHC)

Surgical specimens were immersed in 10% formalin solution for fixation within 30 minutes after dissociation, paraffin-embedded, and sectioned into 4-µm-thick slices. Antigen retrieval was performed using high-pressure heating with 0.01 M citrate buffer (pH 6.0) for 2 minutes. A drop of primary antibody PLOD2 (#A14649, Wuhan Proteintech Biotechnology Co., Ltd.) was added, and the sections were incubated at 4 °C overnight. A drop of biotin-labeled goat anti-mouse/rabbit IgG (#KIT-9720, Cell Signaling Technology, Inc., USA) was added, incubated at room temperature for 10 minutes, followed by color development with a DAB chromogenic kit (#0031/1031, Fuzhou Maixin Biotech Co., Ltd.) for 5 minutes. The results were interpreted independently by two experienced pathologists. To semi-quantitatively evaluate PLOD2 expression, we applied a standard immunoreactive score. Staining intensity was scored as 0 (negative), 1 (weak), 2 (moderate), or 3 (strong), and the percentage of positive cells was scored as 1 (≤25%), 2 (26–50%), 3 (51–75%), or 4 (>75%).The final immunoreactive score was calculated by multiplying the two subscores, yielding a total score ranging from 0 to 12. For statistical analysis, immunoreactive score 0–5 was defined as low expression, and immunoreactive score 6–12 was defined as high expression.

### Cell culture

Caco-2 cells were purchased from Shenzhen Haodi Huatuo Biological Co., Ltd. SW480, SW620, LOVO and HCT116 cells were obtained from the Cell Bank of Chinese Academy of Sciences (Shanghai, China). The cells were cultured at 37 °C and 5% CO_2_^27^, When the density of adherent cells reached 80% (70–90% being appropriate), trypsin (Gibico Inc.) digestion was used for passaging.

### Establishment of cell lines

PLOD2 expression was analyzed in five colorectal cancer cell lines (LOVO, Caco-2, SW620, HCT116, and SW480). Among these, Caco-2 cells exhibited the highest PLOD2 expression, while HCT116 cells showed the lowest. Pre-experiments were conducted using different multiplicities of infection (MOI) to determine the appropriate MOI. Polybrene (final concentration: 10 μg/ml) was added to each well to enhance viral infection of the cells. A lentivirus silencing PLOD2 expression was transfected into the colorectal cancer cell line Caco-2 (Shenzhen Haodi Huatuo Biological Co.), which highly expressed PLOD2. The PLOD2-silenced colorectal cancer cell line was obtained by puromycin selection and named Caco-2-shPLOD2. Additionally, a lentivirus overexpressing PLOD2 was transfected into the colorectal cancer cell line HCT116 (Shanghai Cell Bank), which expressed low levels of PLOD2. The PLOD2-overexpressing colorectal cancer cell line was obtained by puromycin selection and named HCT116-PLOD2. The corresponding empty control cell line was named Vector.

### Cell proliferation

To assess the proliferative capacity of the cells, CCK-8 assays and plate clone formation assays were performed. For the CCK-8 assays, colorectal cancer cells in the logarithmic growth phase were trypsinized, counted, and seeded into 96-well plates (Shanghai Biyuntian Biotechnology Co.) at 1,500–3,000 cells per well. The cells were divided into an empty control group, an experimental group (overexpression/silencing group), and a blank group (no cell inoculation, PBS added). Each group had three replicate wells and was cultured at 37°C. The cell growth rates were measured at 0 h, 24 h, 48 h, 72 h, and 96 h.

### Colony formation assay

In the plate colony formation assay, cells in the logarithmic growth phase were seeded into 6-well plates at 500 cells per well and assigned to either the control group or the overexpression/silencing group. The cells were then cultured at 37 °C for two weeks, with the medium changed every two days. After 14 days, the cells were photographed and counted against a white A4 paper background. The number of clones was counted using ImageJ software, and the experiment was repeated three times.

### Wound healing assay and transwell assays

We performed wound healing experiments to examine the effect of PLOD2 on cell motility. Approximately 2×10^5^~4×10^5^ cells per well were inoculated in 6-well plates and routinely cultured for 48 h. A 200 µL yellow pipette tip was used to create three horizontal scratches per well. The plates were then incubated at 37 °C with 5% CO_2_. Samples were taken at 0 and 72h. Images were captured at each time point to measure the relative width of the wound area, allowing quantification of the wound-healing rate based on cell migration over time.

For the Transwell migration assay, Transwell chambers (Becton, Dickinson and Company) with 8 µm pore size were used, while Matrigel-coated transwell chambers were used for the transwell invasion assay. A 500 µl cell suspension containing 1×10^5^−1.5×10^5^ trypsin-digested colorectal cancer cells was added into the transwell chambers, and the cells were cultured for 24 to 48 h. The cells were then stained with crystal violet. The cells in the sublayer of the microporous membrane were observed under a microscope and photographed. Five random fields of view were taken per well, and ImageJ software was used for cytometry.

### Quantitative realtime PCR

QRT-PCR analyses were performed on a Real-Time PCR instrument (Applied Biosystems, USA) using the PrimeScript RT kit (TaKaRa, Japan). The primer sequences used for PLOD2 were as follows: forward, 5’-GCGTTCTCTTCGTCCTCAT-3’, reverse, 5’-CCACCTCCCTGAAAGTCTTC-3’. Gene expression levels were analyzed using the 2-ΔΔCt method, with GAPDH serving as a constitutive marker^24^, The primer sequences for GAPDH were as follows: forward, 5’-TGCACCACCAACTGCTTAGC −3’, reverse, 5’-AGCTCAGGGATGACCTTGCC −3’.

### Western blotting

The expression of PLOD2 protein was assessed using Western blotting. Cells and tissues were collected and lysed with PMSF (Shanghai Biyuntian Biotechnology Co., Ltd.) and cocktail (Sinopharm Chemical Reagent Co., Ltd.) in RIPA buffer. Cells were lysed on ice for 30 minutes, scraped, and then centrifuged at 12,000 × g for 10 minutes at 4°C. Protein concentrations were determined using the BCA Protein Assay Kit (Beyotime Biotechnology) following the manufacturer’s instructions. Proteins were separated by SDS-PAGE and transferred onto a polyvinylidene difluoride membrane (Bio-Rad Laboratories, USA). The membranes were blocked with 5% bovine serum albumin for 2 h at room temperature. They were then incubated overnight at 4 °C with the primary antibody in the blocking solution. After three washes with TBS containing 0.1% Tween20, the membranes were incubated with the appropriate secondary antibody for 45 minutes at room temperature, followed by three additional washes with TBS. Finally, the PVDF membrane was placed in an ImageQuant LAS4000mini chemiluminescence imager for exposure to capture protein images.

Antibodies used in this experiment were as follows, primary antibody: PLOD2 antibody (#21214-1-AP, Wuhan Proteintech Biotechnology Co., Ltd.), GAPDH antibody (5174,Cell Signaling Technology, Inc., USA), PI3K antibody (4257, Cell Signaling Technology, Inc., USA).Phosoho-PI3K antibody (4228, Cell Signaling Technology, Inc., USA).AKT antibody (4691, Cell Signaling Technology, Inc., USA), Phosoho-AKT antibody (13038, Cell Signaling Technology, Inc., USA). GSK3β antibody (12456, Cell Signaling Technology, Inc., USA), p-GSK3β antibody (5558, Cell Signaling Technology, USA), rabbit anti-IgG monoclonal antibody (7074, Cell Signaling Technology, Inc., USA).

### Cellular intervention to modulate the PI3K signaling pathway

740Y-P (MedChemExpress Biotechnology, USA) was used to activate the PI3K signaling pathway in Caco-2 cells^28^. Complete medium containing 740Y-P was prepared at a concentration of 20 μM by adding 66.6 μl of the stock solution per ml^29^. Cells were cultured in this medium for 48 h in the incubator, and harvested at the specified times. LY294002 (MedChemExpress Biotechnology, USA) was used to inhibit the PI3K signaling pathway in HCT116 cells. LY294002-containing complete medium was prepared at a concentration of 10 mM by adding 10 μl of the stock solution per ml^30^. Preliminary dose-response pre-tests indicate that LY294002 and 740Y-P effectively modulate pathways at this concentration without inducing cytotoxicity. Cells were treated with 10 μM LY294002 for 48 h in the incubator, and collected at the corresponding time points.

### Co- immunoprecipitation (Co-IP)

Protein-protein interactions were assessed using co-immunoprecipitation. Adherent cells were washed with pre-cooled PBS, then lysed using a pre-configured lysis buffer for 30 minutes. After centrifugation at 12,000 × g for 10 minutes at 4 °C, the supernatant containing 1 mg total protein was incubated with primary antibodies and 50% protein A/G agarose beads (Santa Cruz Biotechnology, Inc., USA) together overnight at 4 °C with gentle shaking. The immunocomplexes were collected by centrifugation, washed three times with cold PBS, and eluted by boiling in 2× SDS loading buffer for 10 minutes. The supernatant was used for subsequent Western blot analysis. Under identical conditions, reverse co-immunoprecipitation experiments were performed.

### Statistics analysis

Statistical analysis of data was performed by R language (version: 4.2.2). Graphing was performed by GraphPad Prism 8 (GraphPad Software, USA, version: 9.5.0). Experimental data were tested for normality using the Shapiro–Wilk test and for homogeneity of variance using Levene’s test. Data conforming to normal distribution were analyzed by t-tests or ANOVA. Otherwise, the Kruskal-Wallis rank sum test was used. All statistical analyses were two-sided tests. A *P*-value of < 0.05 was considered statistically significant and was marked with *. *P*-values of < 0.01 and < 0.001 were marked with ** and ***, respectively.

## Results

### PLOD2 was highly expressed in colorectal cancer tissues and its high expression was associated with poor prognosis in colorectal cancer patients

**Fig. 1:**
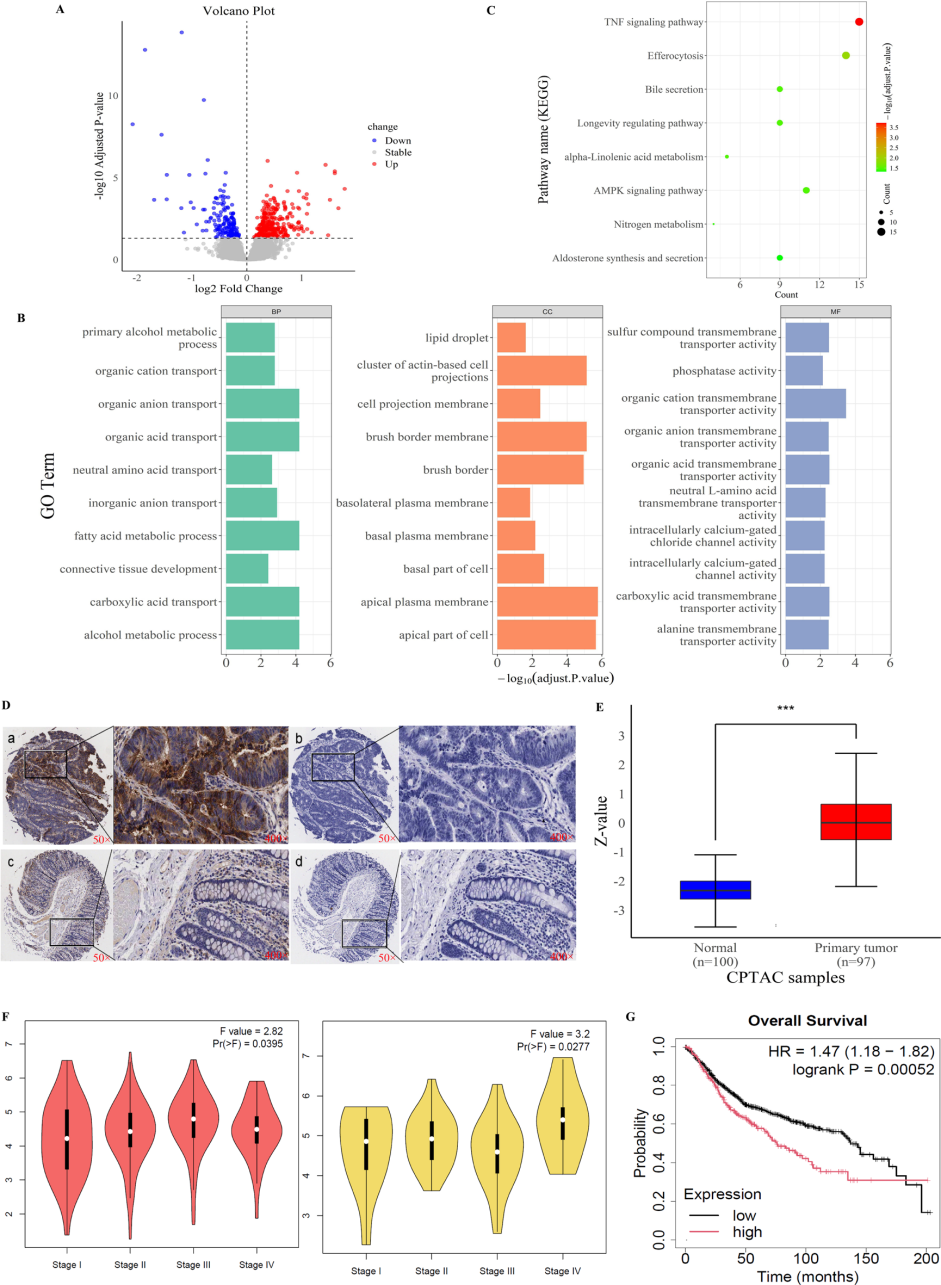
High expression of PLOD2 in Colorectal Cancer. (**A**) Volcano plot showed the DEGs in GSE97689. (**B**) GO enrichment of DEGs and the list of significant GO terms of the top 10. (**C**) KEGG pathways enrichment of DEGs. (**D**) Immunohistochemical detection of PLOD2 expression in colorectal cancer tissues and adjacent normal tissues (a. Strong PLOD2 staining in colorectal cancer tissues; b. Control showing weak PLOD2 staining in colorectal cancer tissues; c. Weak PLOD2 staining in normal tissues adjacent to the tumor; d. Negative control in normal tissues adjacent to the tumor. Images captured at 50× and 400× magnification, respectively.). (**E**) Expression levels of PLOD2 in colorectal cancer and normal tissues from the CPTAC database (z-values denote the standard deviation from the median sample for a given cancer type). (**F**) Correlation of PLOD2 expression with pathological staging of colon and rectal cancer tumors (a, Colon adenocarcinoma; b, Rectal adenocarcinoma.). (**G**) Kaplan-Meier analysis of overall survival in patients with high versus low PLOD2 expression.

Bioinformatics analysis was performed using 92 CRC tissue samples and paired adjacent normal tissues from the GSE97689 dataset. Volcano plot visualization of the GSE126209 dataset revealed differentially expressed genes, with downregulated genes shown in blue and upregulated genes (including PLOD2) in red (Fig. 1 A). GO analysis of DEGs encompassed three categories: biological processes, molecular functions, and cellular components. The most significantly enriched terms included carboxylic acid transport, apical plasma membrane localization, and organic cation transmembrane transporter activity (Fig. 1 B). KEGG pathway analysis identified the TNF signaling pathway as a key pathway among DEGs (Fig. 1 C).

We further validated PLOD2 expression in 75 CRC cases using tissue microarray immunohistochemistry. PLOD2 was predominantly localized in the cytoplasm, showing distinct brownish-yellow or brownish-brown staining (Fig. 1D). The positive expression rate of PLOD2 was 78.67% in CRC tissues compared to 29.33% in adjacent normal tissues, with a significantly higher expression in cancer tissues (Supplementary material). The chi-square test indicated that high PLOD2 expression correlated with CRC stage, lymph node metastasis, and perineural invasion (*P*<0.05), but showed not statistically significant correlation with other clinical characteristics such as age, gender, distant metastasis, differentiation, and lymphovascular invasion (Table 1).

**Table 1:** Correlation analysis between clinicopathological features and PLOD2 expression.

Characteristics	PLOD2 expression		*χ*^2^	*P*-value
	High[N (%)]	Low [N (%)]		
Age (years)			1.739	0.238
<60	15 (48.39)	28 (63.64)		
≥60	16 (51.61)	16 (36.36)		
Gender			0.368	0.641
Male	14 (45.16)	23 (52.27)		
Female	17 (54.84)	21 (47.73)		
Clinical stage			5.526	**0.022**
T1-T2	1 (3.23)	10 (22.73)		
T3-T4	30 (96.77)	34 (77.27)		
Lymph node metastasis			18.725	**<0.001**
N0	3 (9.68)	26 (59.09)		
N+	28 (90.32)	18 (40.91)		
Distant metastasis			3.303	0.153
M0	27 (87.10)	43 (97.73)		
M1	4 (12.90)	1 (2.27)		
Differentiation			—	0.665
Low/undifferentiation	2 (6.45)	2 (4.55)		
High/middle	29 (93.55)	42 (95.45)		
Infiltration			4.016	0.059
superficial muscle layer	9 (29.03)	23 (52.27)		
deep muscular layer	22 (70.97)	21 (47.73)		
Lymphovascular invasion			—	1.000
Absent	24 (77.42)	35 (79.55)		
Present	7 (22.58)	9 (20.45)		
Nerve invasion			4.962	**0.039**
Absent	21 (67.74)	39 (88.64)		
Present	10 (32.26)	5 (11.36)		

We analyzed protein expression data from the CPTAC dataset using the UALCAN online database, and the results showed significantly upregulated PLOD2 protein expression in CRC (*P*<0.001) (Fig. 1E). GEPIA2’s "Pathological Stage Plot" module indicated that PLOD2 expression fluctuated across pathological stages in colorectal cancer, with the highest values observed in stage III colon cancer and stage IV rectal cancer. However, pairwise comparisons between stages did not reach statistical significance (Fig. 1F). Moreover, using the Kaplan-Meier Plotter database’s Colorectal Cancer Survival Curve Plotting Module, the results showed that among 1055 CRC patients, those with high PLOD2 expression had a lower overall survival rate compared to those with low expression (*P*<0.05) (Fig. 1G).

### PLOD2 promoted colorectal cancer cell proliferation, migration and invasion

**Fig. 2:**
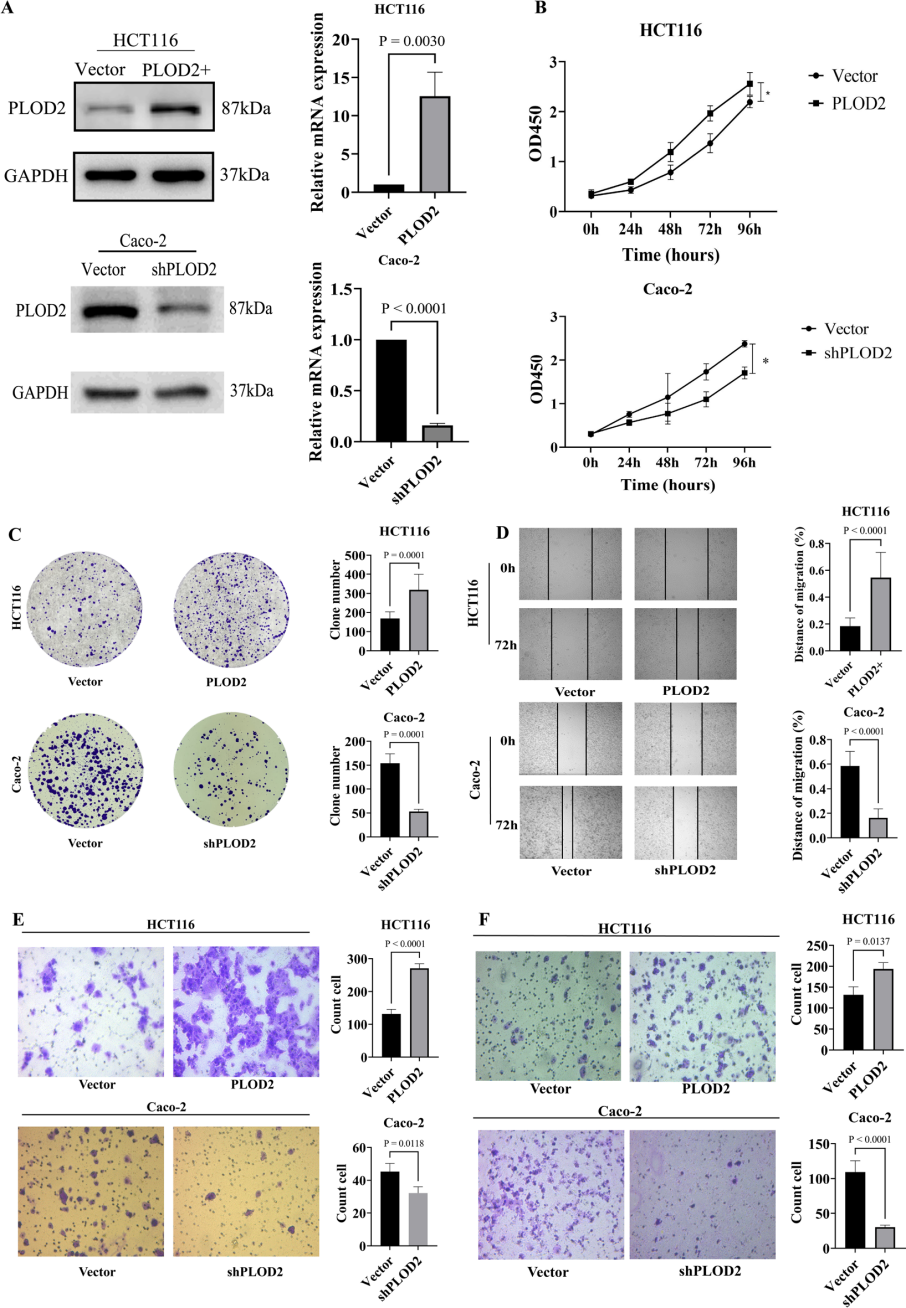
PLOD2 promotes cell proliferation, migration, and invasion in CRC cells. (**A**) Western blot and qPCR analysis of PLOD2 protein and mRNA expression in HCT116-PLOD2 and Caco-2-shPLOD2 cell lines, uncropped gel and blot images with edges marked are presented in Supplementary material. (**B**) Effect of PLOD2 on the proliferative capacity of colorectal cancer cells, assessed by CCK-8 assay. (**C**) Effect of PLOD2 on colony formation and proliferative ability of colorectal cancer cells, assessed by plate colony formation assay. (**D**) Effect of PLOD2 on the horizontal migration ability of colorectal cancer cells, assessed by cell Wound healing assay. (**E**) Effect of PLOD2 on the vertical migration ability of colorectal cancer cells, assessed by Transwell migration assay. (**F**) Effect of PLOD2 on the invasive ability of colorectal cancer cells, assessed by Transwell invasion assay.

Western blot results showed that in the HCT116-PLOD2 cell line, the protein and mRNA expression levels of PLOD2 were significantly higher than those in control cells. In the Caco-2-shPLOD2 cell line, the protein and mRNA expression levels of PLOD2 were significantly lower than those in control cells, indicating that the construction of colorectal cancer cell lines with stable overexpression and silenced expression of PLOD2 was successful (Fig. 2A).

The results of the CCK-8 assay showed that the OD450 values of HCT116-PLOD2 cells were significantly higher than those of the control group, and those of Caco-2-shPLOD2 cells were significantly lower than those of the control group, indicating that PLOD2 promotes the proliferation of colorectal cancer cells (Fig. 2B). The plate clone formation assay was used to evaluate the clone formation ability and proliferation ability of colorectal cancer cells (Fig. 2C). The wound healing assay was used to evaluate the horizontal migration of colorectal cancer cells (Fig. 2D), and the transwell assay assessed both the vertical migration ability (Fig. 2E) and invasive ability (Fig. 2F) of colorectal cancer cells. The results showed that PLOD2 overexpression in HCT116-PLOD2 cells enhanced proliferation, migration, and invasion, whereas PLOD2 knockdown in Caco-2-shPLOD2 cells attenuated these abilities.

### PLOD2 activated the PI3K-AKT-GSK3β signaling pathway in colorectal cancer cells

**Fig. 3:**
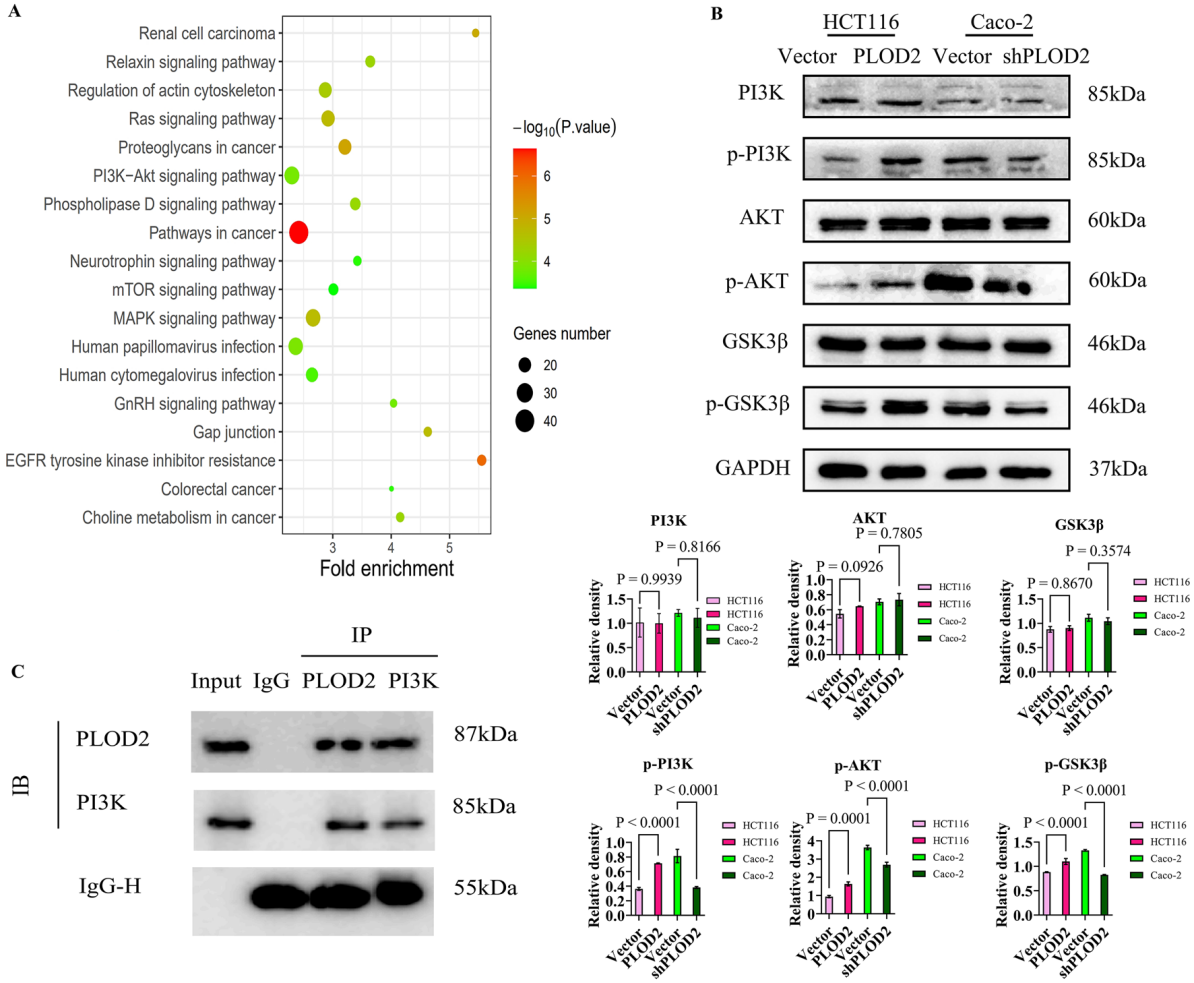
PLOD2 activates the PI3K-AKT-GSK3β signaling pathway in colorectal cancer cells. (**A**) KEGG enrichment analysis of PLOD2-interacting proteins and 500 expression-related genes. (**B**) Effects of PLOD2 overexpression and silencing on PI3K-AKT-GSK3β signaling pathway proteins and expression quantification, uncropped gel and blot images with edges marked are presented in Supplementary material. (**C**) Co-immunoprecipitation experiments for PLOD2 and PI3K, uncropped gel and blot images with edges marked are presented in Supplementary material.

To explore the potential molecular mechanisms of PLOD2 in colorectal carcinogenesis, GEPIA2 was used to analyze the expression data of colon and rectal cancers in the TCGA database to identify the top 500 genes associated with PLOD2 expression. These 50 PLOD2-interacting proteins and 500 expression-related genes were included in the DAVID database for KEGG pathway enrichment analysis. The results indicated that PLOD2 might influence colorectal cancer progression through the "PI3K-AKT signaling pathway" (Fig. 3A).

Based on the results of KEGG enrichment analysis, we hypothesized that PLOD2 might influence colorectal cancer progression through the PI3K–AKT signaling pathway. GSK3β, a key downstream target of this pathway, was known to be involved in multiple aspects of tumor progression. Western blot analysis showed that the levels of phosphorylated PI3K, AKT, and GSK3β were higher in HCT116-PLOD2 cells than in control cells, whereas their levels were lower in Caco-2-shPLOD2 cells compared with controls. The total levels of PI3K, AKT, and GSK3β remained largely unchanged in both groups (Fig. 3B). These results suggested that PLOD2 expression might be associated with activation of the PI3K–AKT–GSK3β signaling pathway.

Co-immunoprecipitation analysis further showed that in HCT116-PLOD2 cells, immunoprecipitation with a PI3K-specific antibody pulled down a complex containing PLOD2 and PI3K, as compared with the positive control (Input) and negative control (IgG) (Fig. 3C). Similarly, immunoprecipitation with a PLOD2-specific antibody resulted in the co-precipitation of PI3K, confirming a physical interaction between PLOD2 and PI3K. These findings suggest that PLOD2 may regulate the PI3K–AKT–GSK3β signaling pathway through its association with PI3K.

### PLOD2 promoted colorectal cancer cell proliferation, migration and invasion through the PI3K-AKT-GSK3β signaling pathway

**Fig. 4:**
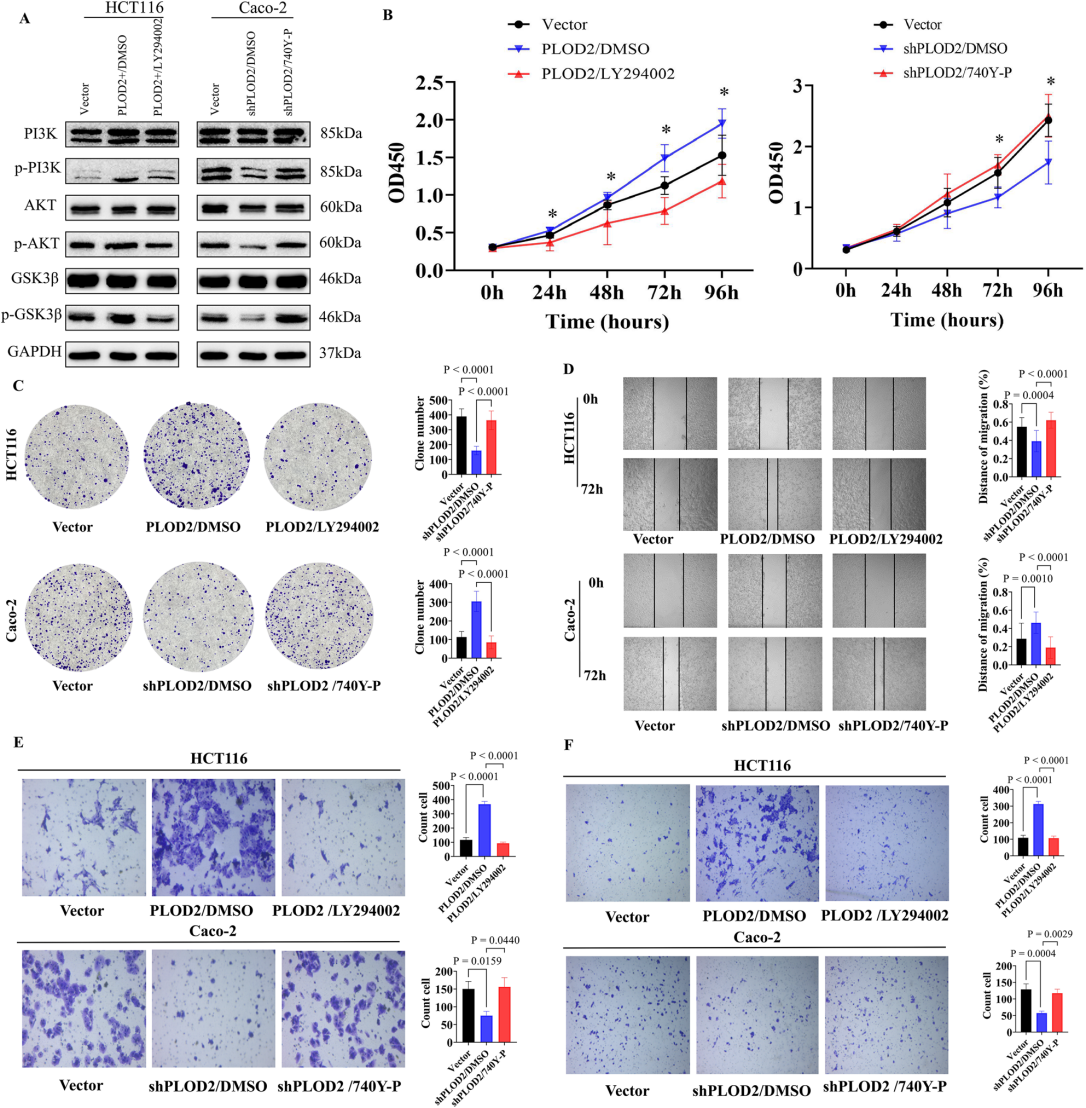
PLOD2 promotes CRC cell proliferation, migration, and invasion through the PI3K-AKT-GSK3β signaling pathway. (**A**) Expression of PI3K-AKT-GSK3β pathway-related proteins in colorectal cancer cells treated with the PI3K inhibitor (LY294002, 10 μM) or agonist (740Y-P, 20 μM) for 48 h, uncropped gel and blot images with edges marked are presented in Supplementary material. (**B**) CCK-8 assay assessing the proliferative capacity of colorectal cancer cells treated with PI3K inhibitors or agonists. (**C**) Colony formation assay evaluating the proliferation of colorectal cancer cells treated with PI3K inhibitors or agonists. (**D**) Wound healing assay evaluating the horizontal migration ability of colorectal cancer cells treated with PI3K inhibitors or agonists. (**E**) Transwell migration assay assessing the vertical migration ability of colorectal cancer cells treated with PI3K inhibitors or agonists. (**F**) Transwell invasion assay evaluating the invasive ability of colorectal cancer cells treated with PI3K inhibitors or agonists.

To confirm that PLOD2 regulated colorectal cancer cell progression by activating the PI3K-AKT-GSK3β signaling pathway through its interaction with PI3K, rescue experiments were conducted using the PI3K inhibitor and the agonist on HCT116-PLOD2 and Caco-2-shPLOD2 cell lines, respectively. Western blot analysis showed significantly increased levels of p-PI3K, p-AKT, and p-GSK3β in HCT116-PLOD2 cells, with no significant changes in total PI3K, AKT, and GSK3β protein levels (Fig. 4A). The PI3K inhibitor was able to suppressed the expression levels of p-PI3K, p-AKT, and p-GSK3β in HCT116-PLOD2 cells. Conversely, in Caco-2-shPLOD2 cells, p-PI3K, p-AKT, and p-GSK3β levels were significantly decreased, with no significant changes in total PI3K, AKT, and GSK3β protein levels. The PI3K agonist was able to restore p-PI3K, p-AKT, and p-GSK3β levels in Caco-2-shPLOD2 cells. These results demonstrate that PLOD2 activates the PI3K-AKT-GSK3β signaling pathway by interacting with PI3K.

To further confirm the mechanism by which PLOD2 affected the functional alterations of colorectal cancer cells, a series of cellular function rescue experiments were performed by adding PI3K inhibitors and agonists to HCT116-PLOD2 and Caco-2-shPLOD2 cell lines, respectively. The results showed that the proliferative, clone forming, migratory, vertical migratory, and invasive abilities of HCT116-PLOD2 cells were inhibited after the addition of PI3K inhibitors. Conversely, the proliferation, clone formation, migration, vertical migration, and invasion abilities of Caco-2-shPLOD2 cells were restored after the addition of PI3K agonists (Fig. 4B-F). Both LY294002 and 740Y-P significantly reversed the PLOD2-mediated effects on proliferation, migration, and invasion. Although the reversal markedly reduced the PLOD2-induced phenotypes, the rescue was partial rather than complete, indicating that PLOD2 may exert additional modulatory effects beyond PI3K-AKT signaling.

## Discussion

As a key enzyme in forming stable collagen cross-links, PLOD2 plays a crucial role in the stability of the tumor cell mesenchyme^31^. Numerous studies have reported that overexpression of PLOD2 is significantly associated with cancer metastasis and poor prognosis in various cancer types, including lung cancer, breast cancer, and glioma^16,17,20^. In our study, we used bioinformatics analysis to discover that PLOD2 is highly expressed in colorectal cancer tissues. In vitro experiments revealed that PLOD2 promotes the proliferation, migration, and invasion of colorectal cancer cells. Jiawen Lan et al. found that PLOD2 promotes colorectal cancer progression by activating the AKT-mTOR signaling pathway through stabilization of USP15^24^, which is consistent with our findings. This provides a valuable mechanistic comparison and further supports the importance of upstream signaling regulators—such as PLOD2—in driving CRC aggressiveness through pathway-level activation. Furthermore, recent clinical evidence shows that inflammation- and nutrition-related biomarkers serve as important predictors of CRC prognosis and treatment response^32^. These findings highlight the importance of integrating tumor-intrinsic molecular drivers like PLOD2 with systemic host-associated biomarkers to achieve more comprehensive prognostic assessment. In addition, IL27RA-mediated immune regulation revealed by single-cell transcriptomic profiling^10^ demonstrates that microenvironmental immune signals critically influence CRC progression, supporting the broader relevance of our findings on ECM- and signaling-driven tumor regulation.

To investigate the potential molecular mechanism of PLOD2 in colorectal carcinogenesis, we employed bioinformatic analysis. The results indicate that PLOD2 may affect colorectal cancer progression through the "PI3K-AKT signaling pathway". PI3K is an intracellular lipid kinase responsible for the phosphorylation of various enzymes, which is crucial in cellular functions and cancer development^33^. AKT, a major downstream target of PI3K, controls cell proliferation, survival, and the cell cycle^34^. The PI3K-AKT signaling pathway plays an important role in normal cellular activity, and its dysfunction can lead to various diseases, including diabetes and autoimmune diseases^35,36^. Although KEGG analysis of DEGs in the GSE97689 dataset showed significant enrichment of the TNF signaling pathway, we focused our mechanistic investigation on the PI3K–AKT pathway. This decision was guided not only by the DEG results but also by the KEGG enrichment of PLOD2-interacting proteins, which prominently highlighted the PI3K–AKT pathway and therefore suggested a more direct functional link to PLOD2 than the TNF pathway.Moreover, accumulating evidence indicates that PLOD2 promotes tumor progression through extracellular matrix remodeling, EMT, and enhanced cell motility^37,38^—processes predominantly regulated by PI3K–AKT–GSK3β signaling rather than TNF-mediated inflammation. Activation of PI3K–AKT leads to GSK3β phosphorylation and inactivation, subsequent stabilization of β-catenin, and induction of EMT and invasive behavior in colorectal cancer cells. These well-characterized mechanisms closely align with the phenotypic changes observed in our PLOD2 gain- and loss-of-function models. Therefore, the PI3K–AKT pathway was selected for validation because it represents the most biologically plausible and well-supported mechanistic axis linking PLOD2 to colorectal cancer progression.

AKT activation phosphorylates its downstream targets, such as MDM2, TSC2, GSK3, FOXO, and mTOR^39–41^, to regulate a series of biological activities, including cell growth, survival, proliferation, and glucose metabolism. These processes are involved in the occurrence and development of cancers, cardiovascular diseases, diabetes mellitus, and neurological diseases. The PI3K-AKT signaling pathway plays a role in various cancers, including breast, liver, and pancreatic cancers^36,42–44^. Additionally, during colorectal cancer development, mutations in PIK3CA and PIK3CB, AKT mutation or amplification, PTEN loss of function, and mTORC1 overactivation can activate the PI3K–AKT signaling pathway. This activation may contribute to the malignant transformation of benign lesions^45^. Overexpression of AKT also exacerbates colon cancer progression along with the PI3K signaling pathway^46–48^. GSK3β is a proline-directed serine/threonine protein kinase involved in energy metabolism and neuronal cell development. It serves as a negative regulator of glucose homeostasis and plays a role in energy metabolism, inflammation, endoplasmic reticulum stress, mitochondrial dysfunction, and apoptosis^49,50^. Abnormal expression of GSK3β has been linked to several diseases, including cancer, type 2 diabetes, cardiovascular diseases, and neurodegenerative disorders. GSK3β is an important downstream target of the PI3K-AKT signaling pathway, with its activity inhibited upon phosphorylation by AKT, thereby regulating cellular metabolic processes^51^. The PI3K-AKT-GSK3β signaling pathway has been shown to contribute to the progression of colorectal cancer^52–54^, which is consistent with the findings of our study.

In our study, we found that overexpression of PLOD2 increased the levels of p-PI3K, p-AKT, and p-GSK3β in colorectal cancer cells, while silencing PLOD2 decreased their levels. In contrast, the total expression of PI3K, AKT, and GSK3β remained unchanged. PI3K inhibitors and agonists could reverse the promotion and inhibition effects caused by overexpression and silencing of PLOD2. These findings suggest that PLOD2 does not affect the intrinsic expression levels of PI3K, AKT, and GSK3β but regulates the progression of colorectal cancer by activating the PI3K-AKT-GSK3β signaling pathway. Phosphorylation of GSK3β by AKT leads to its inactivation, preventing it from phosphorylating β-catenin. This results in a massive accumulation of β-catenin in the cytoplasm, which then enters the nucleus and activates genes related to cell division and growth regulation^55^. In addition to regulating β-catenin stability, GSK3β plays several important roles in colorectal cancer biology. First, GSK3β is a key suppressor of EMT; its inactivation promotes EMT progression and enhances tumor cell migration and invasion^56^. Second, GSK3β helps maintain the degradation of multiple oncogenic transcription factors, such as Snail and c-Myc^57^, and its inhibition therefore contributes to a more aggressive phenotype. Third, GSK3β is involved in metabolic reprogramming by modulating glycogen metabolism and mitochondrial homeostasis, processes that are frequently hijacked by cancer cells to support rapid growth^58^. Taken together, phosphorylation-mediated inactivation of GSK3β may represent an important mechanism by which PLOD2 activates PI3K–AKT signaling to enhance proliferation, migration, invasion, and metabolic adaptation in colorectal cancer. Notably, recent mechanistic studies have shown that signaling-driven malignant behaviors in CRC can also be promoted by oncogenic regulators such as GNL3L, which activates NF-κB signaling to enhance tumor growth and metastasis^59^.

To date, relatively few studies have investigated how PLOD2 regulates colorectal cancer progression. Although PLOD2 has been reported to promote colorectal cancer progression by activating the AKT/mTOR signaling pathway through stabilization of USP15^24^, it is well established that the mTOR pathway primarily regulates cell growth and metabolism. In this study, we demonstrate for the first time that PLOD2 promotes colorectal cancer cell proliferation, migration, and invasion by activating the PI3K–AKT–GSK3β signaling pathway. These results indicate that GSK3β acts as a key downstream effector in PLOD2-mediated tumor metastasis. An important novelty of our study is the identification of a direct physical interaction between PLOD2 and PI3K. This finding extends the current understanding of PLOD2-mediated signaling beyond the previously reported USP15–AKT/mTOR axis. Our co-immunoprecipitation assays confirmed that PLOD2 binds directly to PI3K, suggesting that PLOD2 may function as an upstream regulator that facilitates PI3K activation. This mechanistic insight provides a new conceptual framework for understanding how PLOD2 drives CRC progression and may offer additional therapeutic targets. Compared with previous studies, our findings provide deeper insights into the novel mechanisms by which PLOD2 contributes to malignant tumor progression and further expand the current understanding of its oncogenic roles.

Several limitations should be acknowledged in this study. First, most of the mechanistic findings were obtained from in vitro experiments using a limited number of CRC cell lines, which may not fully reflect the complexity of tumor behavior in vivo. Second, although we identified an association between PLOD2 and activation of the PI3K–AKT–GSK3β signaling pathway, causal relationships were not directly confirmed by in vivo functional assays or animal models. Third, clinical data were analyzed retrospectively and were obtained from a relatively small cohort, which may introduce selection bias and limit the generalizability of our findings. Finally, while our results suggest that PLOD2 has potential as a prognostic or therapeutic biomarker, further validation in large-scale, prospective, and multicenter clinical studies is required to substantiate its clinical applicability.

## Conclusions

Our study provides evidence that PLOD2 is upregulated in CRC and is associated with increased CRC cell proliferation, migration, and invasion. The results suggest that PLOD2 may influence these processes, at least in part, through the modulation of the PI3K–AKT–GSK3β signaling pathway. Collectively, these findings indicate that PLOD2 has potential as a prognostic or therapeutic biomarker for CRC, although further validation in future clinical studies is warranted.

## Supplementary Information

Supplementary Information.

## Data Availability

The genomic sequencing data and associated datasets analyzed in this study were obtained from publicly available repositories, including the Gene Expression Omnibus (GEO), The Cancer Genome Atlas (TCGA), UALCAN, and the European Genome-phenome Archive (EGA).

## Abbreviations

CRC: Colorectal cancer
PLOD2: Procollagen-lysine, 2-oxoglutarate 5-dioxygenase 2
KEGG: Kyoto encyclopedia of genes and genomes
ECM: Extracellular matrix
EMT: Epithelial–mesenchymal transition
DEGs: Differentially expressed genes
GEO: Gene expression omnibus
GO: Gene ontology
TCGA: The cancer genome atlas
IHC: Immunohistochemistry
CO-IP: Co- immunoprecipitation
