# Supplementary material for: PLOD2 promotes proliferation, migration and invasion of colorectal cancer cells via PI3K-AKT-GSK3β signaling pathway
Source: Sci Rep. 2026 Feb 10;16:8118. doi: 10.1038/s41598-026-38593-6 (PMC12960814; doi:10.1038/s41598-026-38593-6)
Supplement: Supplementary file 1 — Supplementary Information. [file 41598_2026_38593_MOESM1_ESM.pdf]

Table S1: Immunohistochemical detection of PLOD2 protein expression in tissues

**Tissues**	**PLOD2 Expression Score**							
	Low		High					
	**3**	**4**	**6**	**8**	**9**	**12**	χ^2	***P***
**cancerous**	12	4	19	24	8	12	50.49	<0.001
**adjacent normal**	22	31	10	12	0	0		

Figure 2A

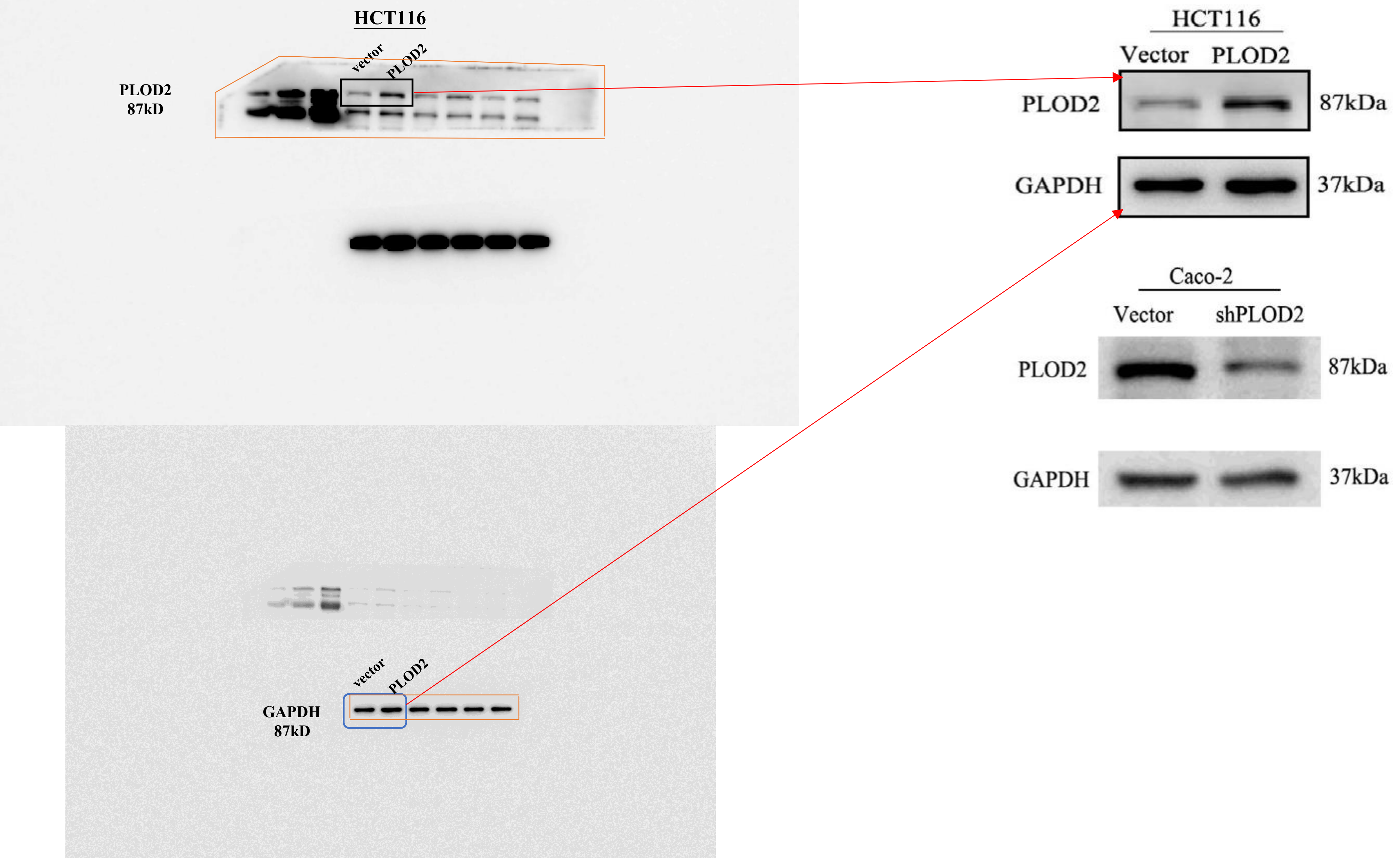

HCT116
vector
PLOD2
PLOD2
87kD
GAPDH
87kD
HCT116
Vector
PLOD2
PLOD2
87kDa
GAPDH
37kDa
Caco-2
Vector
shPLOD2
PLOD2
87kDa
GAPDH
37kDa

Figure 2A

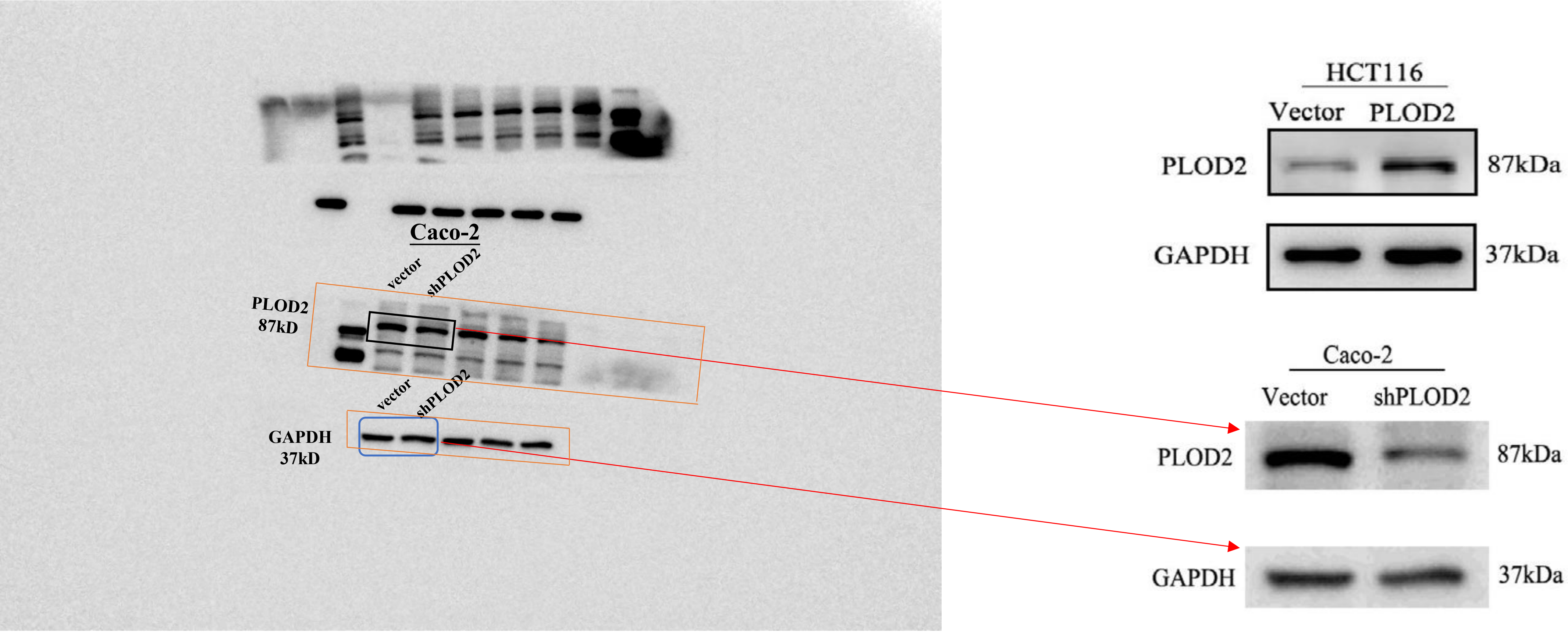
Caco-2
vector
shPLOD2
PLOD2
87kD
vector
shPLOD2
GAPDH
37kD
HCT116
Vector
PLOD2
PLOD2
87kDa
GAPDH
37kDa
Caco-2
Vector
shPLOD2
PLOD2
87kDa
GAPDH
37kDa

Figure 3B

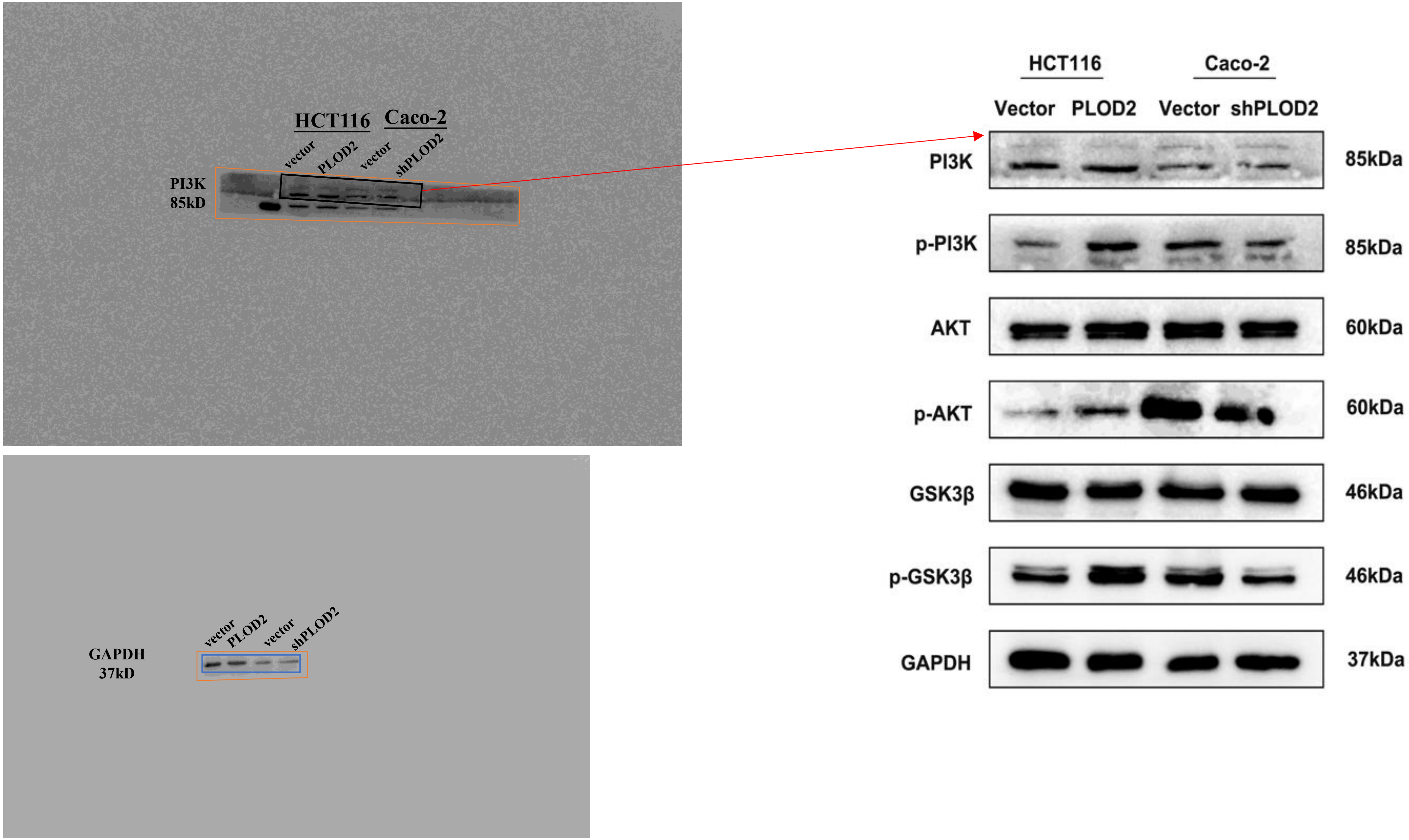
HCT116
Caco-2
vector
PLOD2
vector
shPLOD2
PI3K
85kD
GAPDH
37kD
vector
PLOD2
vector
shPLOD2
HCT116
Caco-2
Vector
PLOD2
Vector
shPLOD2
PI3K
85kDa
p-PI3K
85kDa
AKT
60kDa
p-AKT
60kDa
GSK3β
46kDa
p-GSK3β
46kDa
GAPDH
37kDa

Figure 3B

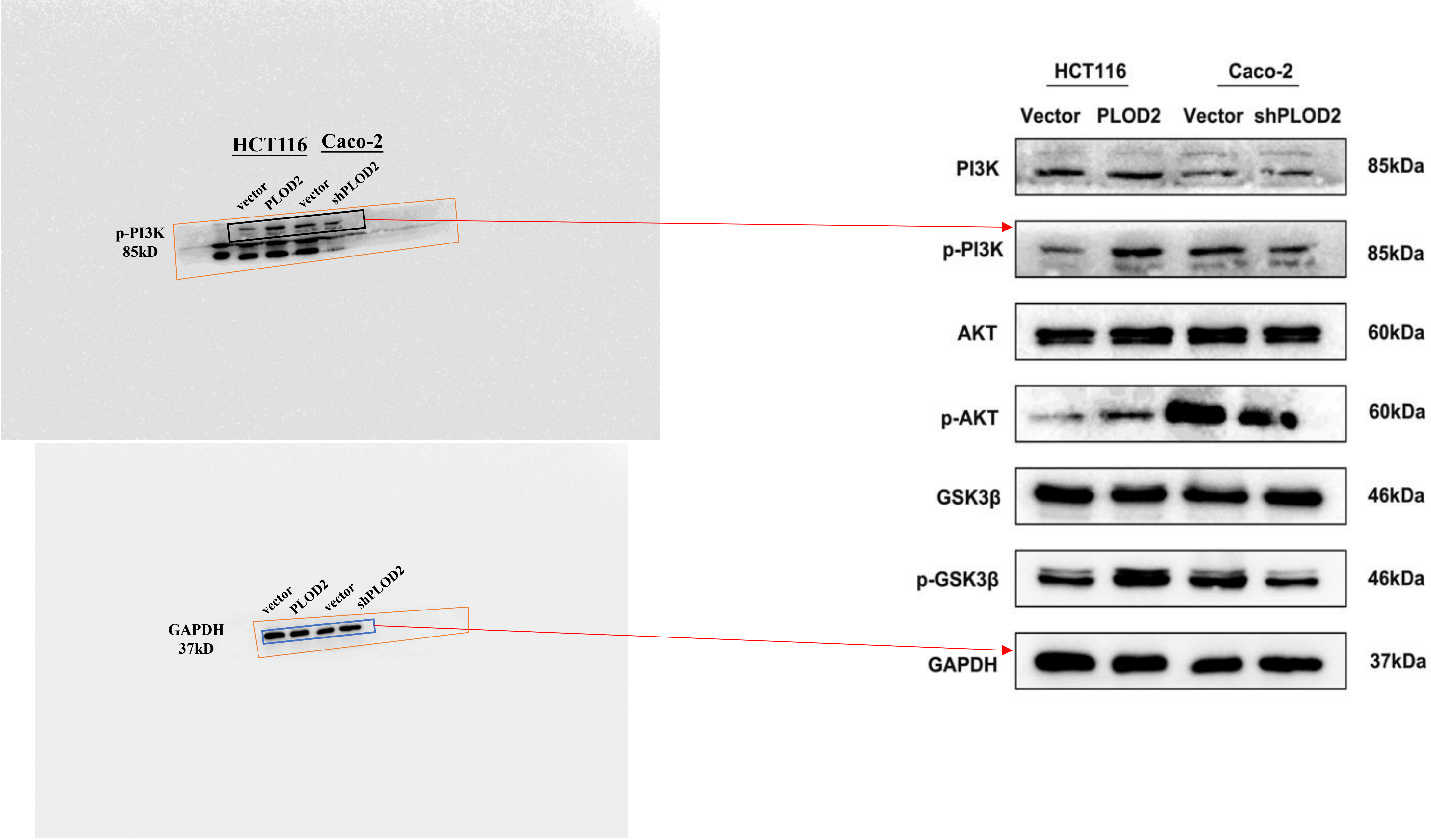

HCT116
Caco-2
vector
PLOD2
vector
shPLOD2
p-PI3K
85kD
GAPDH
37kD
HCT116
Caco-2
Vector
PLOD2
Vector
shPLOD2
PI3K
85kDa
p-PI3K
85kDa
AKT
60kDa
p-AKT
60kDa
GSK3β
46kDa
p-GSK3β
46kDa
GAPDH
37kDa

Figure 3B

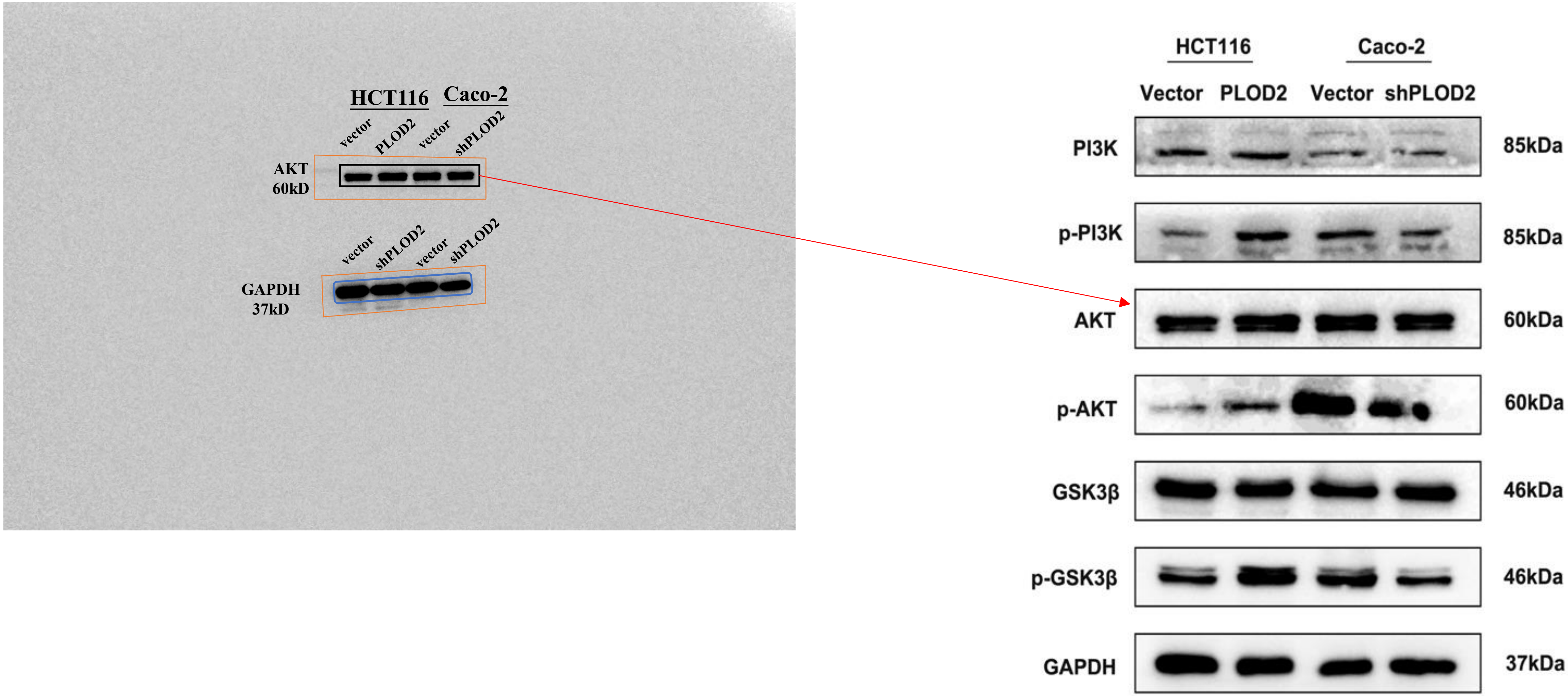

HCT116
Caco-2
vector
PLOD2
vector
shPLOD2
AKT
60kD
vector
shPLOD2
vector
shPLOD2
GAPDH
37kD
HCT116
Caco-2
Vector
PLOD2
Vector
shPLOD2
PI3K
85kDa
p-PI3K
85kDa
AKT
60kDa
p-AKT
60kDa
GSK3β
46kDa
p-GSK3β
46kDa
GAPDH
37kDa

Figure 3B

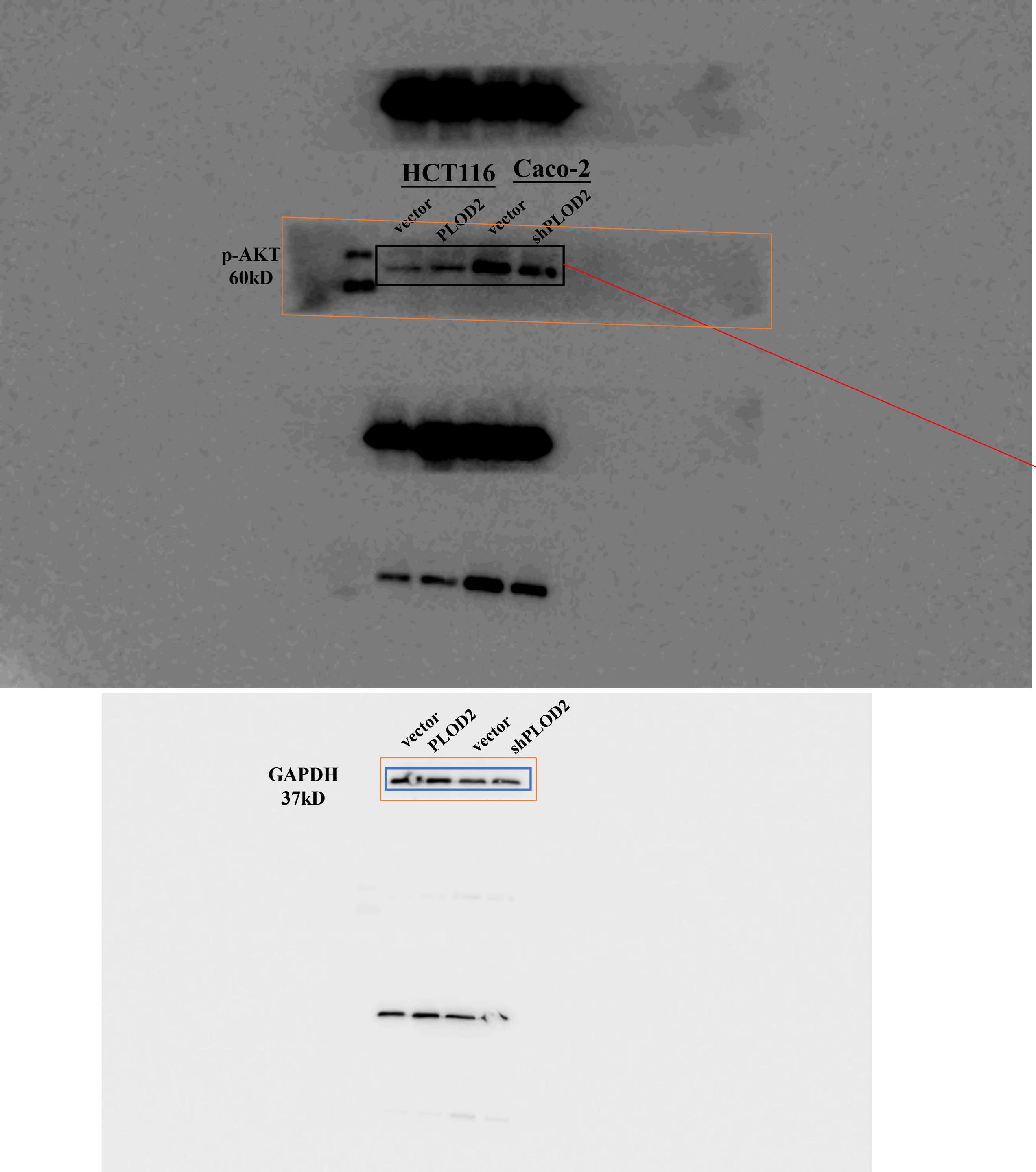
HCT116
Caco-2
vector
PLOD2
vector
shPLOD2
p-AKT
60kD
vector
PLOD2
vector
shPLOD2
GAPDH
37kD

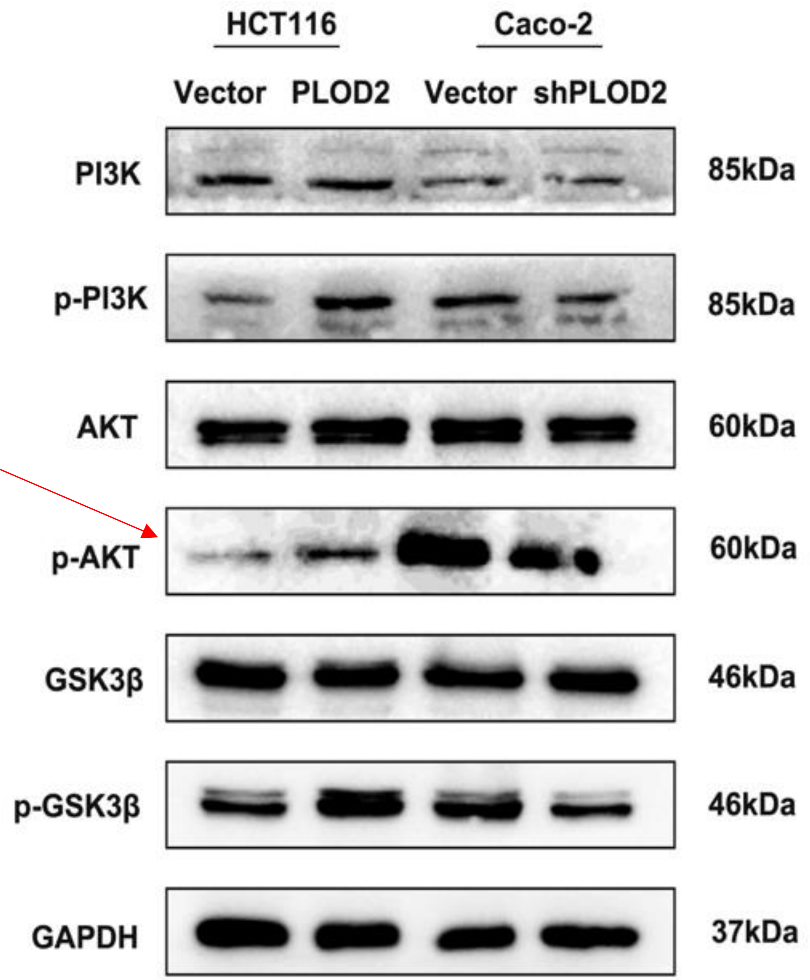
HCT116
Caco-2
Vector
PLOD2
Vector
shPLOD2
PI3K
85kDa
p-PI3K
85kDa
AKT
60kDa
p-AKT
60kDa
GSK3β
46kDa
p-GSK3β
46kDa
GAPDH
37kDa

Figure 3B

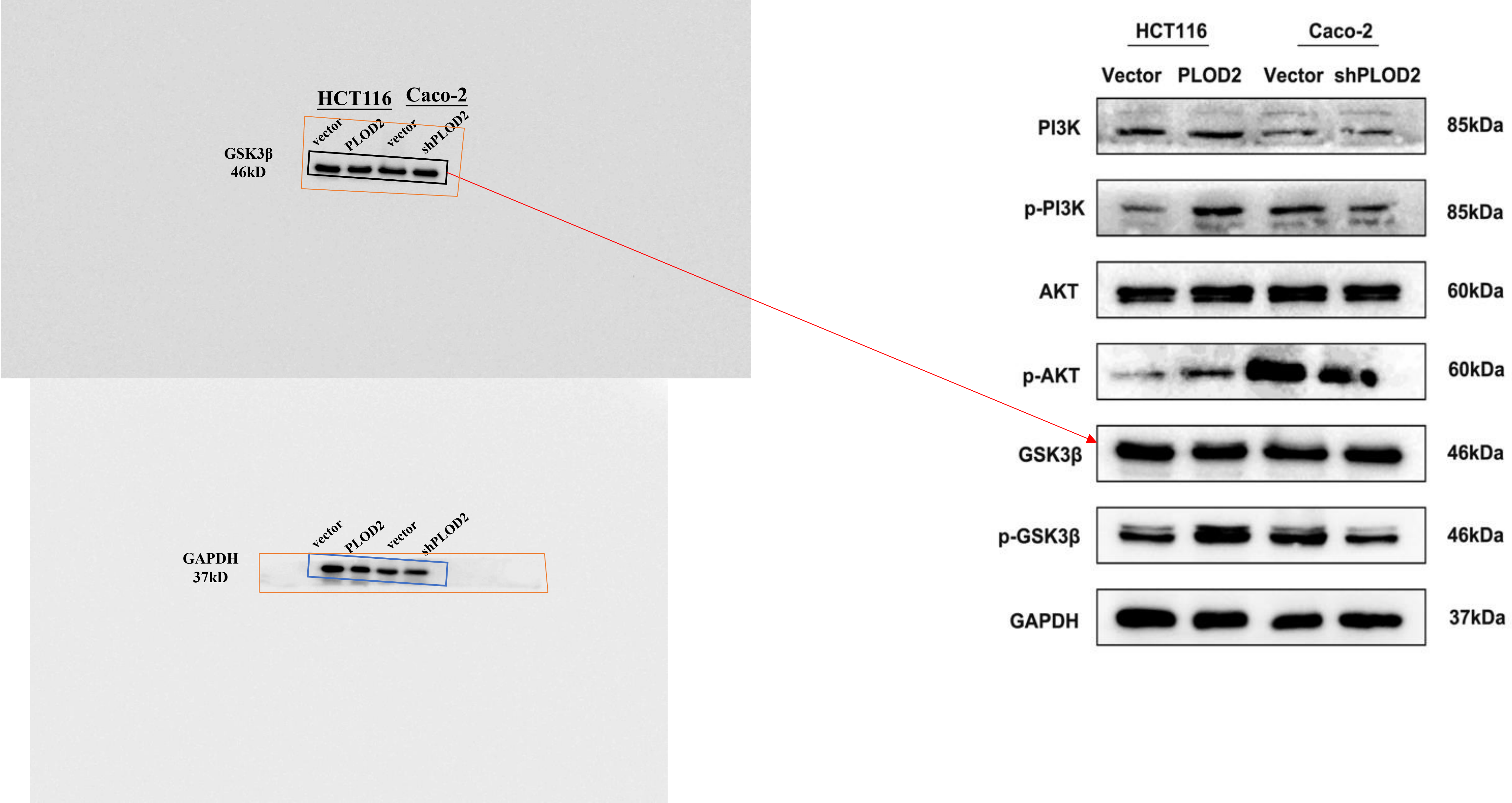
HCT116
Caco-2
vector
PLOD2
vector
shPLOD2
GSK3β
46kD
vector
PLOD2
vector
shPLOD2
GAPDH
37kD
HCT116
Caco-2
Vector
PLOD2
Vector
shPLOD2
PI3K
85kDa
p-PI3K
85kDa
AKT
60kDa
p-AKT
60kDa
GSK3β
46kDa
p-GSK3β
46kDa
GAPDH
37kDa

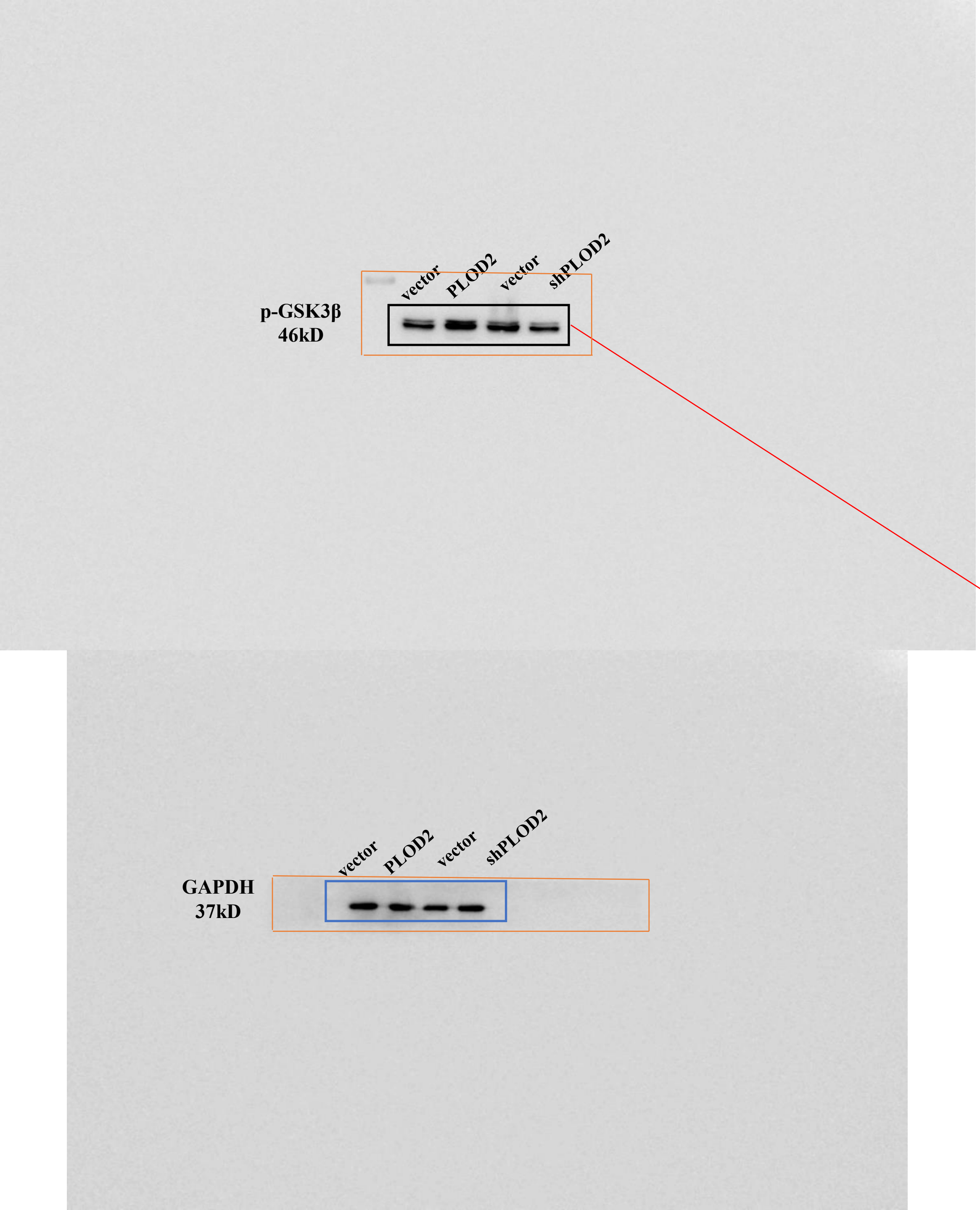
vector
PLOD2
vector
shPLOD2
p-GSK3β
46kD
vector
PLOD2
vector
shPLOD2
GAPDH
37kD

Figure 3B

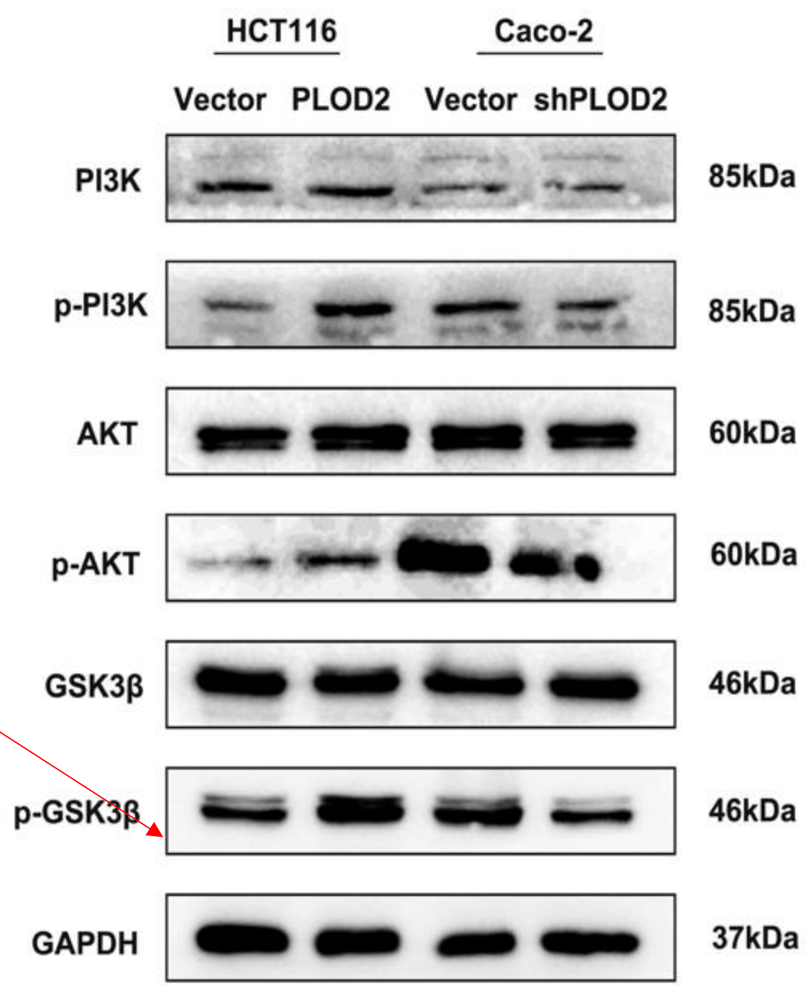
HCT116
Caco-2
Vector
PLOD2
Vector
shPLOD2
PI3K
85kDa
p-PI3K
85kDa
AKT
60kDa
p-AKT
60kDa
GSK3β
46kDa
p-GSK3β
46kDa
GAPDH
37kDa

Figure 3C

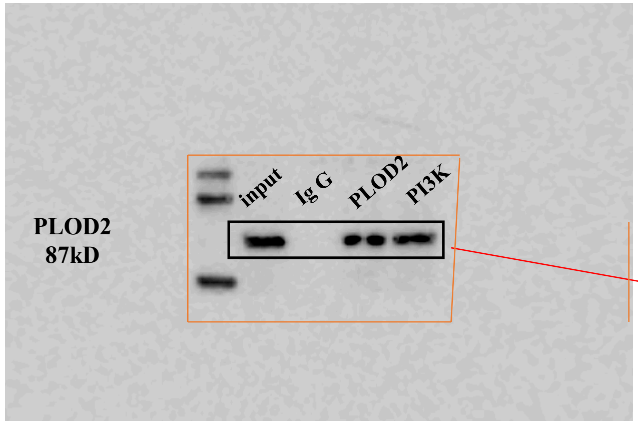
PLOD2
87kD
input
Ig G
PLOD2
PI3K

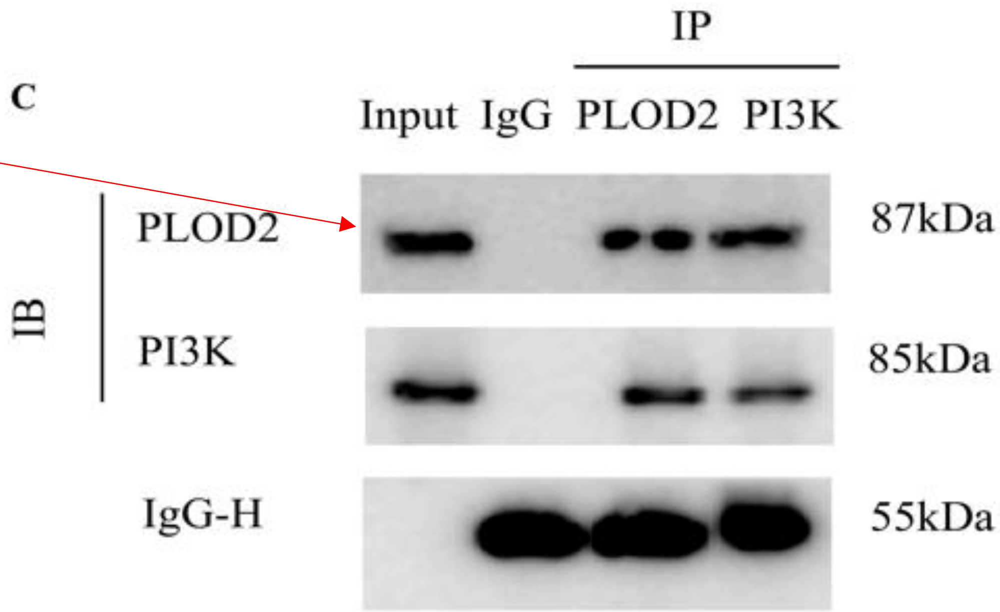
C
IP
Input
IgG
PLOD2
PI3K
IB
PLOD2
87kDa
PI3K
85kDa
IgG-H
55kDa

Figure 3C

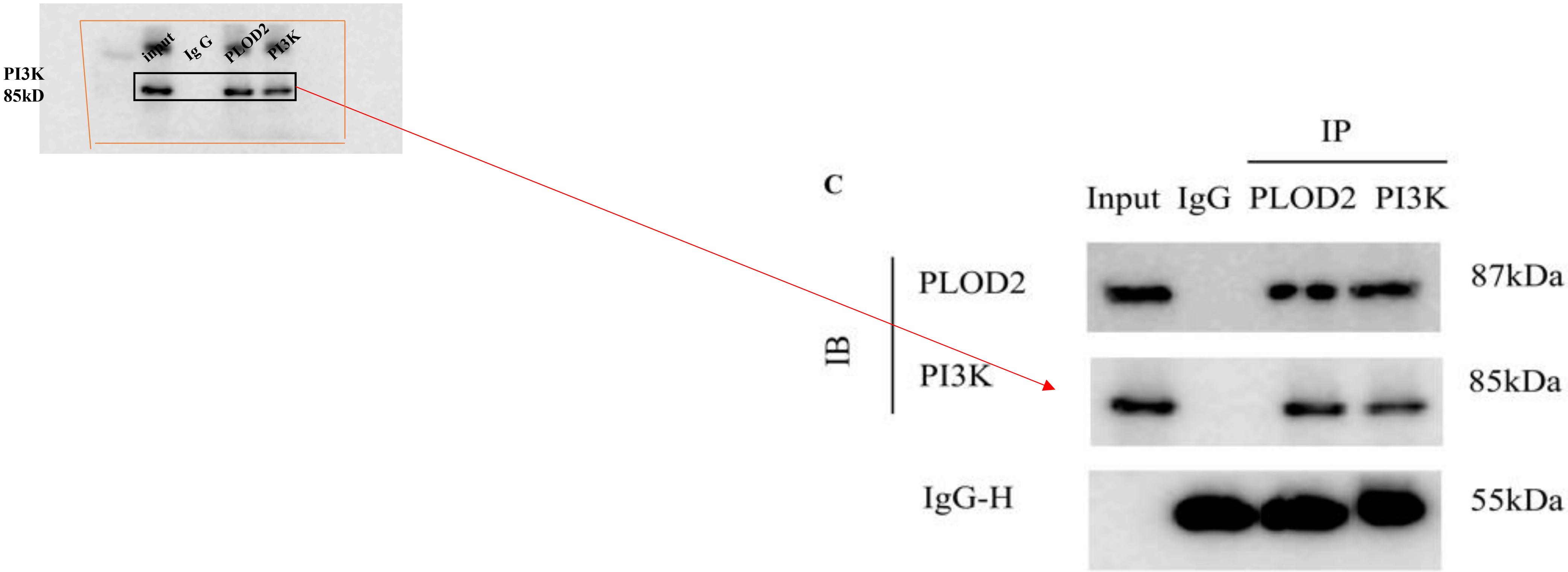
PI3K
85kD
input
Ig G
PLOD2
PI3K
C
IP
Input
IgG
PLOD2
PI3K
IB
PLOD2
PI3K
IgG-H
87kDa
85kDa
55kDa

Figure 3C

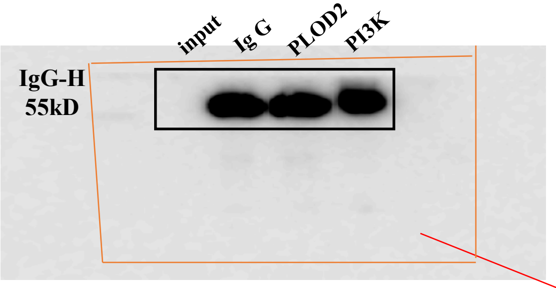
input
Ig G
PLOD2
PI3K
IgG-H
55kD

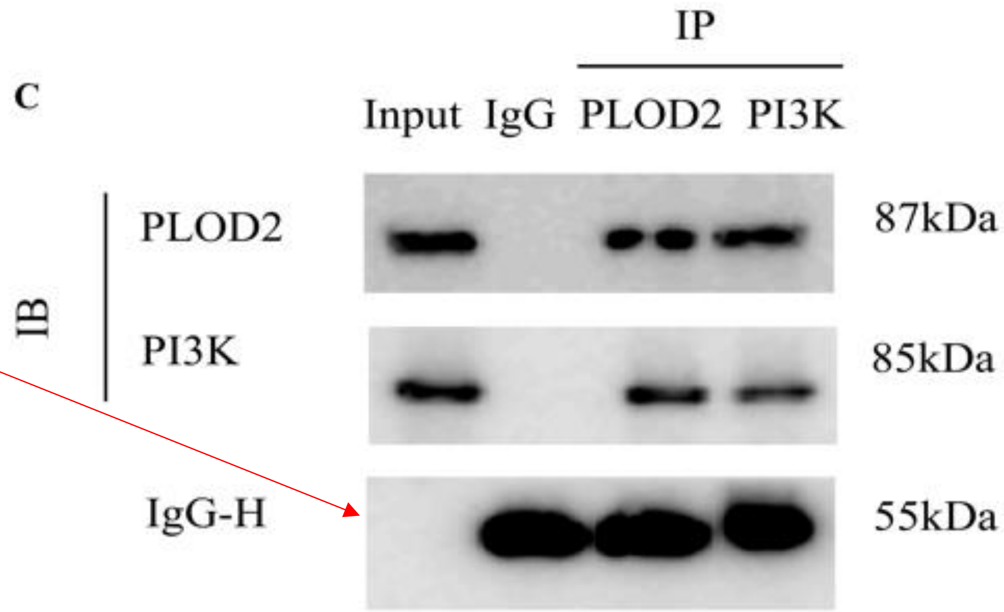
C
IP
Input
IgG
PLOD2
PI3K
IB
PLOD2
87kDa
PI3K
85kDa
IgG-H
55kDa

Figure 4A

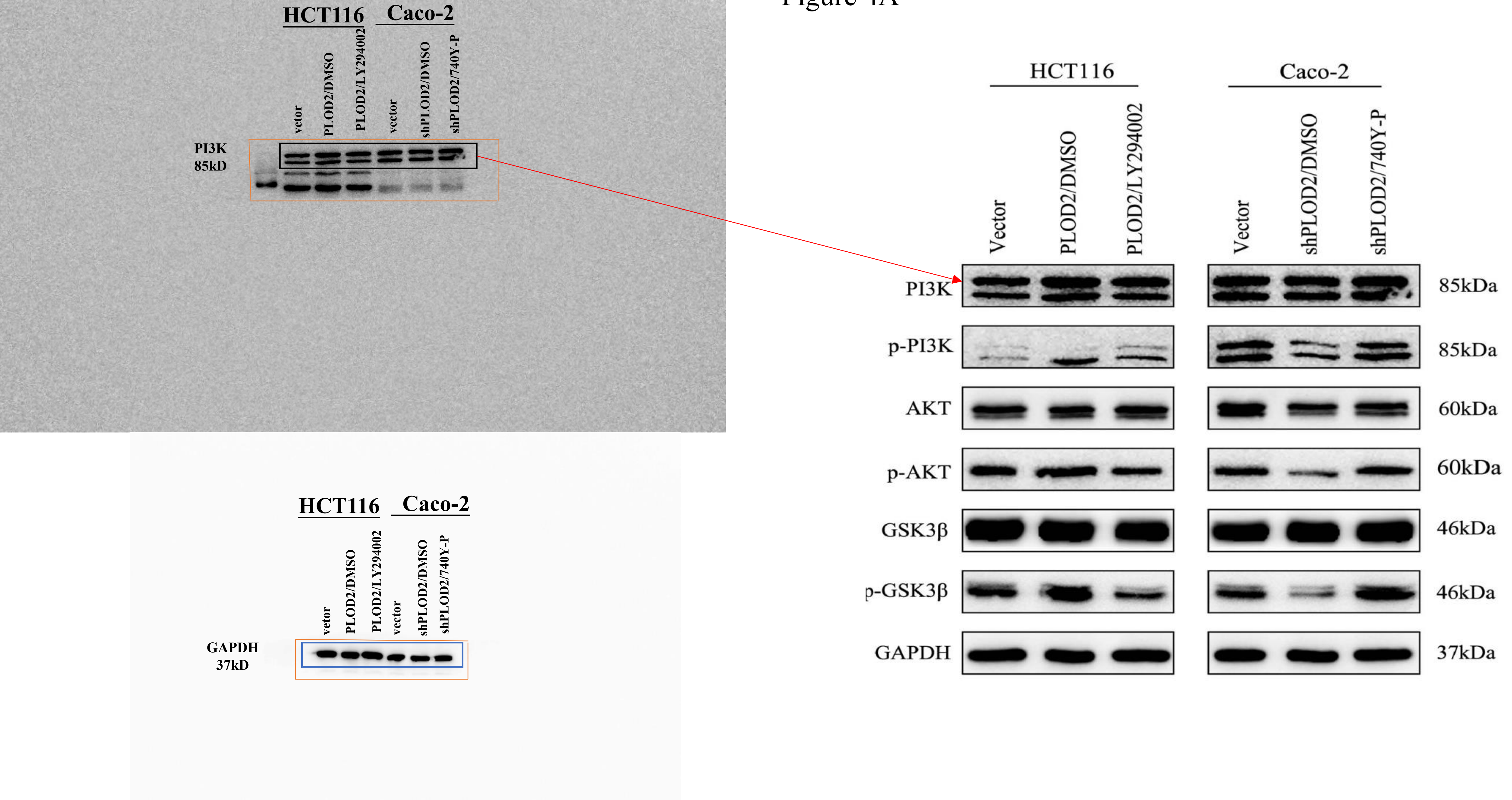
HCT116
Caco-2
vetor
PLOD2/DMSO
PLOD2/LY294002
vector
shPLOD2/DMSO
shPLOD2/740Y-P
PI3K
85kD
GAPDH
37kD
Vector
PLOD2/DMSO
PLOD2/LY294002
Vector
shPLOD2/DMSO
shPLOD2/740Y-P
PI3K
p-PI3K
AKT
p-AKT
GSK3β
p-GSK3β
GAPDH
85kDa
85kDa
60kDa
60kDa
46kDa
46kDa
37kDa

Figure 4A

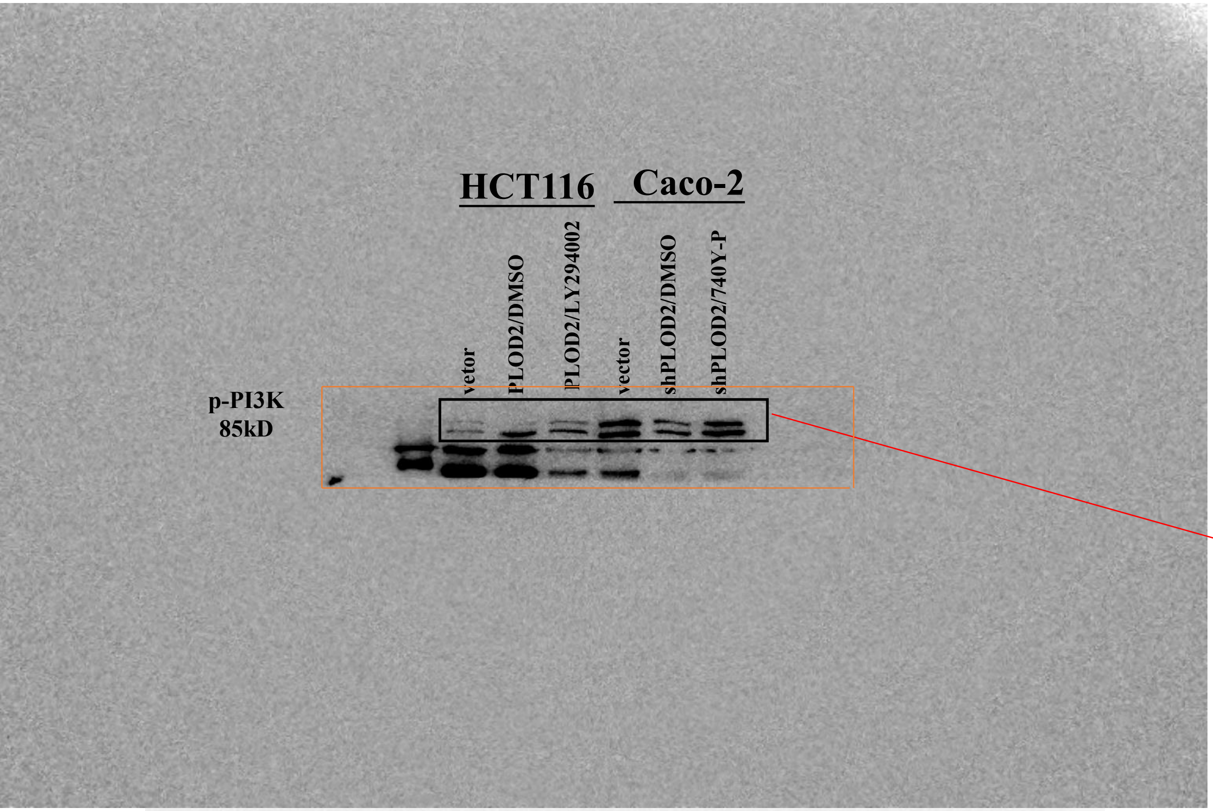

HCT116
Caco-2
vetor
PLOD2/DMSO
PLOD2/LY294002
vector
shPLOD2/DMSO
shPLOD2/740Y-P
p-PI3K
85kD

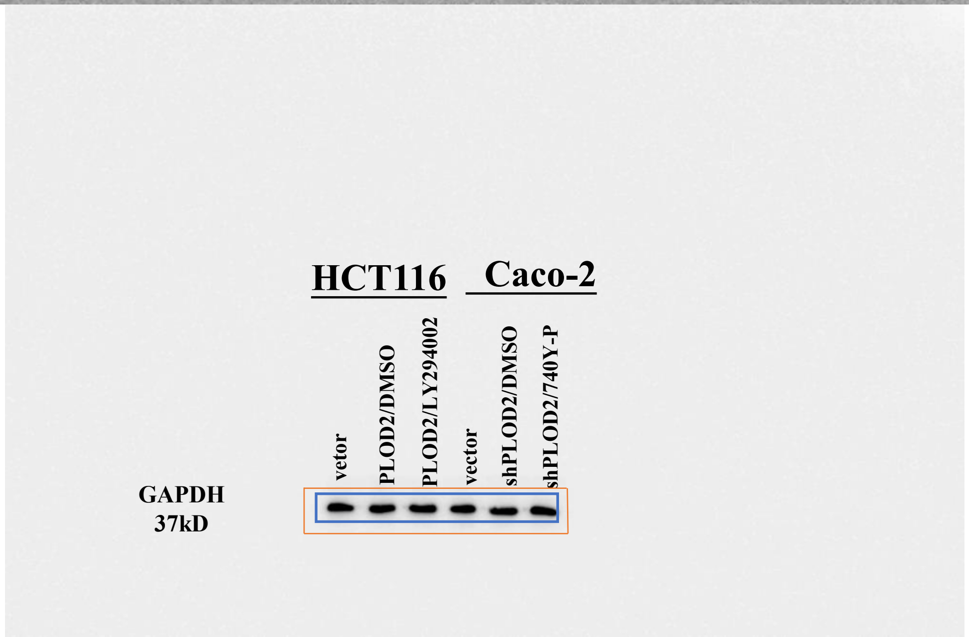

HCT116
Caco-2
vetor
PLOD2/DMSO
PLOD2/LY294002
vetor
shPLOD2/DMSO
shPLOD2/740Y-P
GAPDH
37kD

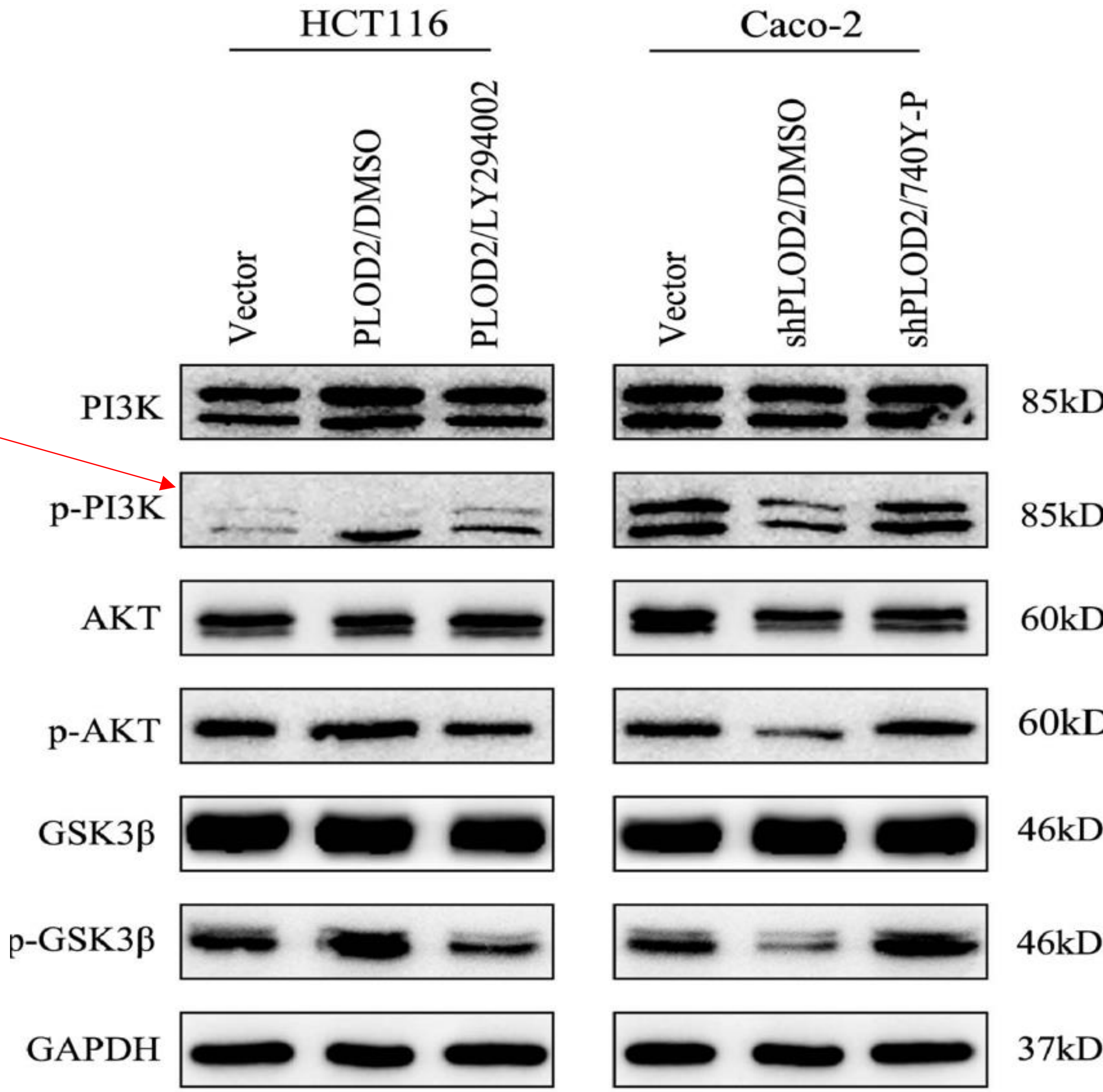

HCT116
Caco-2
Vector
PLOD2/DMSO
PLOD2/LY294002
Vector
shPLOD2/DMSO
shPLOD2/740Y-P
PI3K
p-PI3K
AKT
p-AKT
GSK3β
p-GSK3β
GAPDH
85kD
85kD
60kD
60kD
46kD
46kD
37kD

Figure 4A

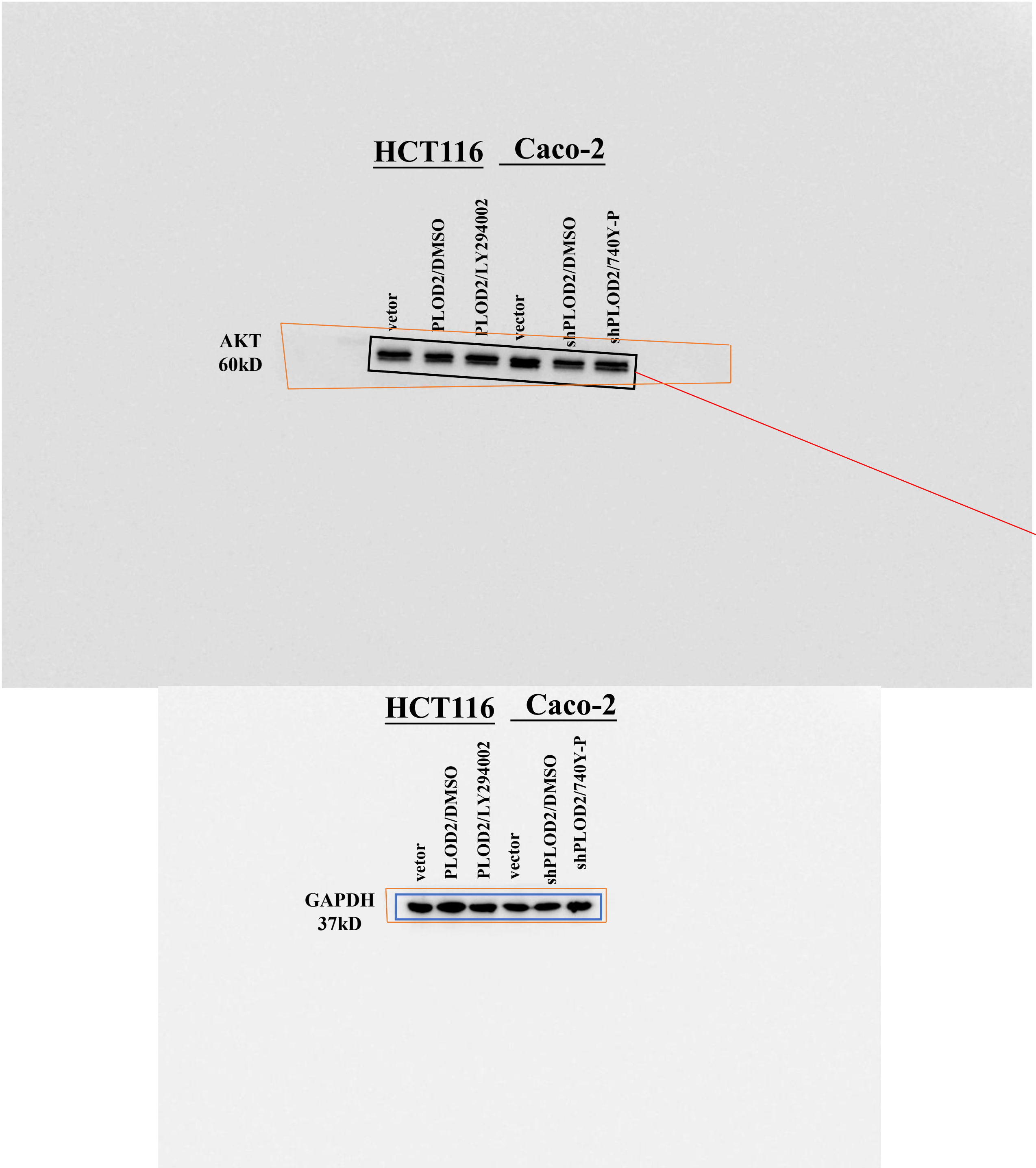

HCT116
Caco-2
vetor
PLOD2/DMSO
PLOD2/LY294002
vector
shPLOD2/DMSO
shPLOD2/740Y-P
AKT
60kD
HCT116
Caco-2
vetor
PLOD2/DMSO
PLOD2/LY294002
vector
shPLOD2/DMSO
shPLOD2/740Y-P
GAPDH
37kD

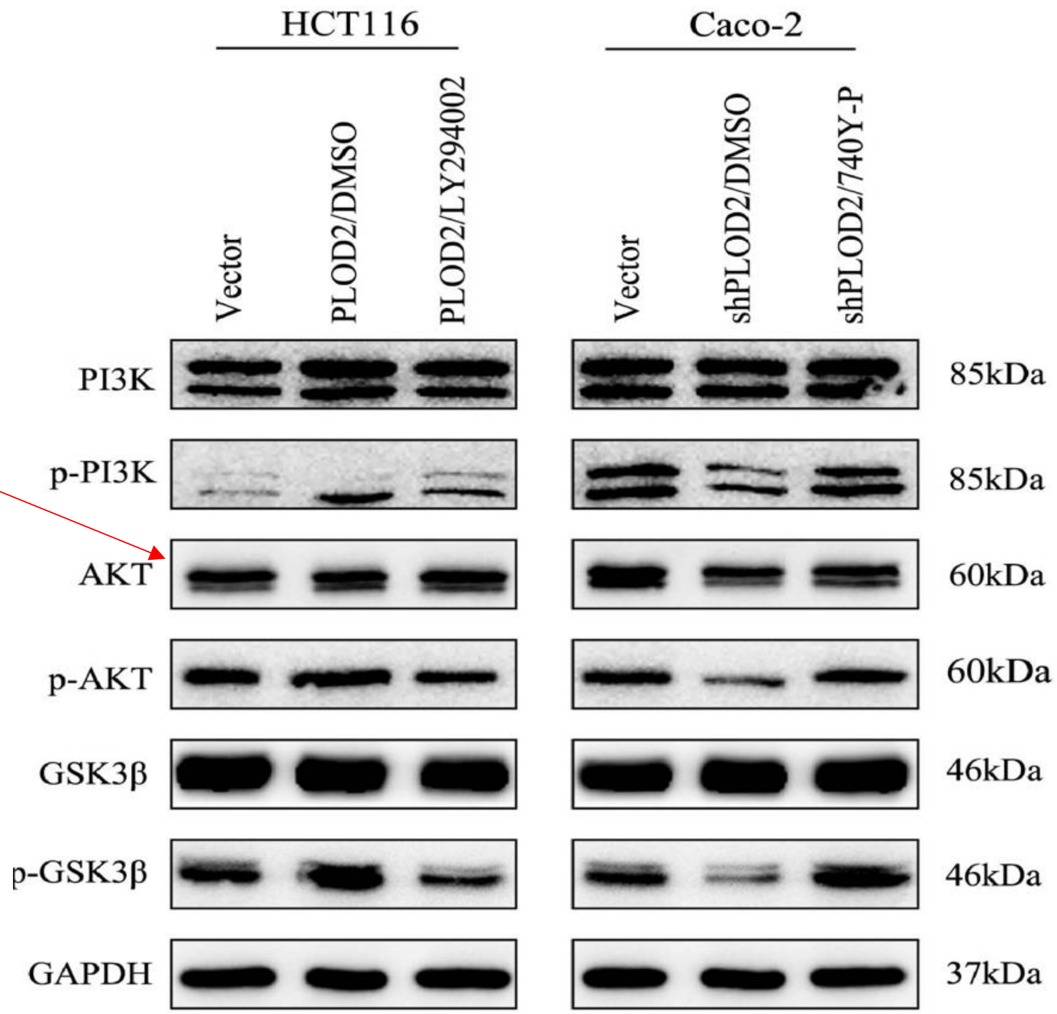

HCT116
Caco-2
Vector
PLOD2/DMSO
PLOD2/LY294002
Vector
shPLOD2/DMSO
shPLOD2/740Y-P
PI3K
85kDa
p-PI3K
85kDa
AKT
60kDa
p-AKT
60kDa
GSK3β
46kDa
p-GSK3β
46kDa
GAPDH
37kDa

Figure 4A

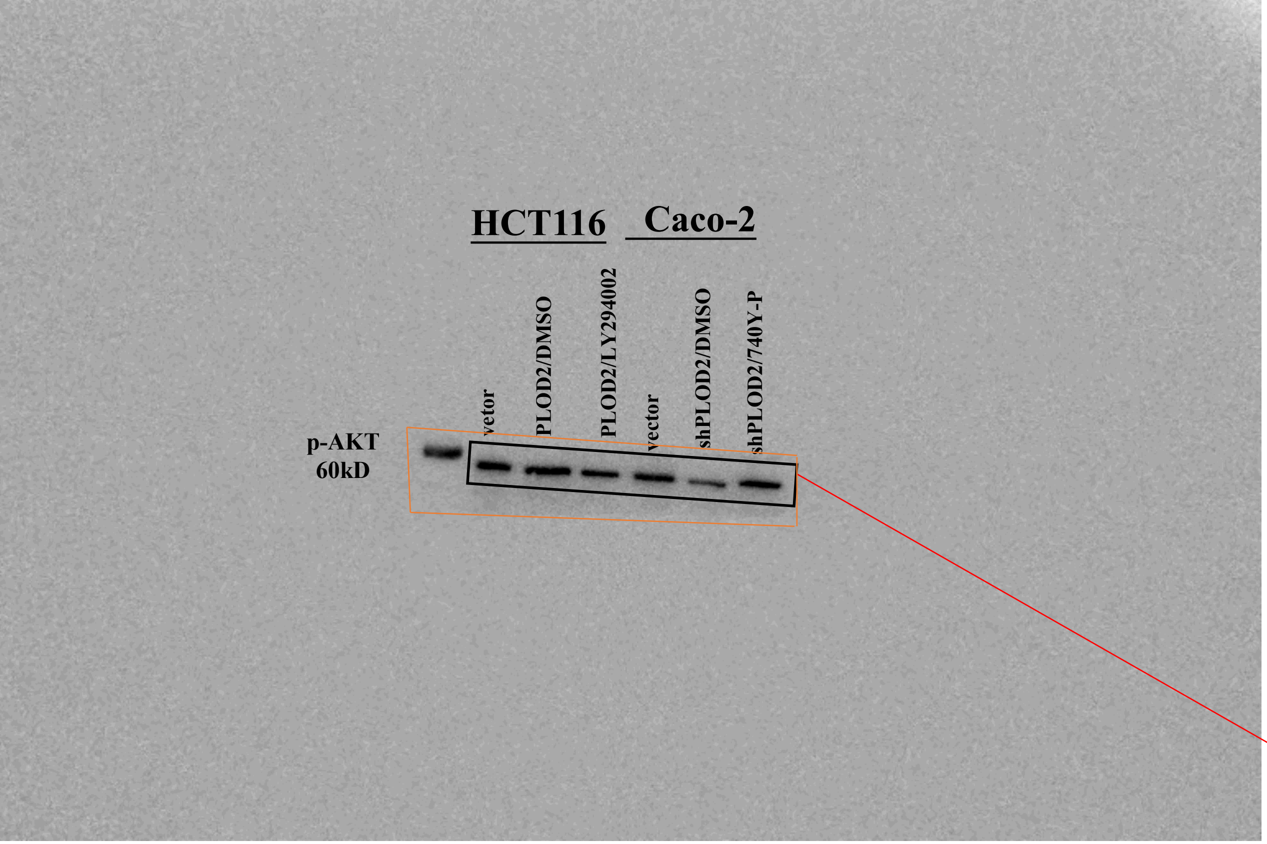
HCT116
Caco-2
vetor
PLOD2/DMSO
PLOD2/LY294002
vector
shPLOD2/DMSO
shPLOD2/740Y-P
p-AKT
60kD

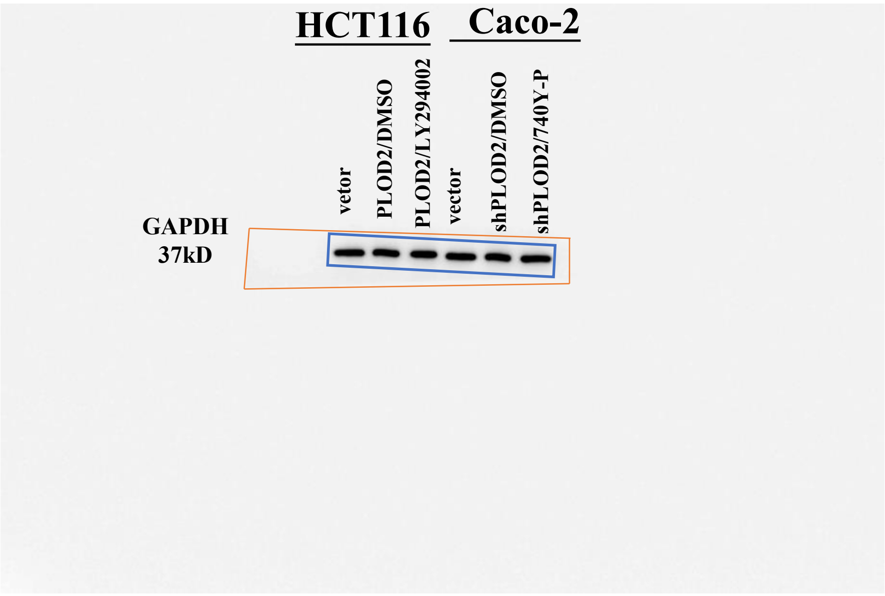
HCT116
Caco-2
vetor
PLOD2/DMSO
PLOD2/LY294002
vector
shPLOD2/DMSO
shPLOD2/740Y-P
GAPDH
37kD

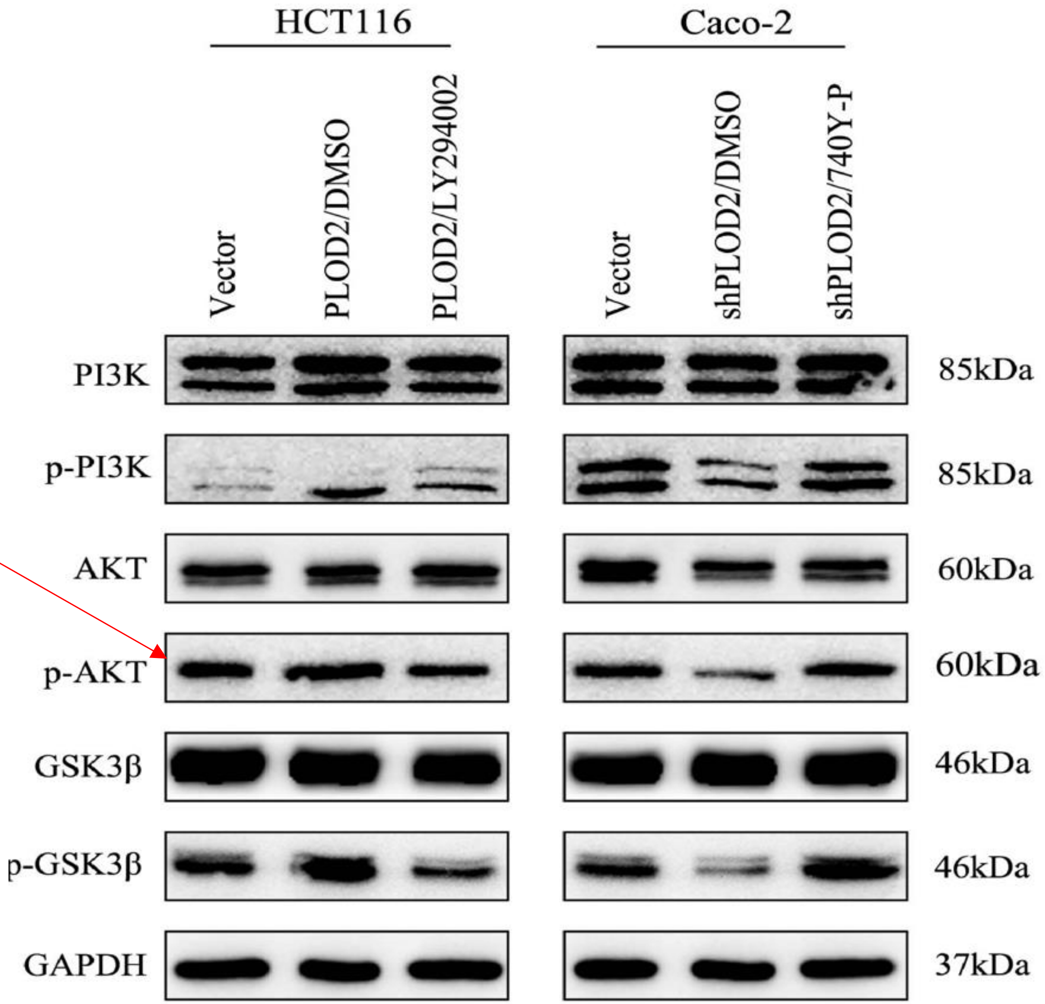
HCT116
Caco-2
Vector
PLOD2/DMSO
PLOD2/LY294002
Vector
shPLOD2/DMSO
shPLOD2/740Y-P
PI3K
85kDa
p-PI3K
85kDa
AKT
60kDa
p-AKT
60kDa
GSK3β
46kDa
p-GSK3β
46kDa
GAPDH
37kDa

Figure 4A

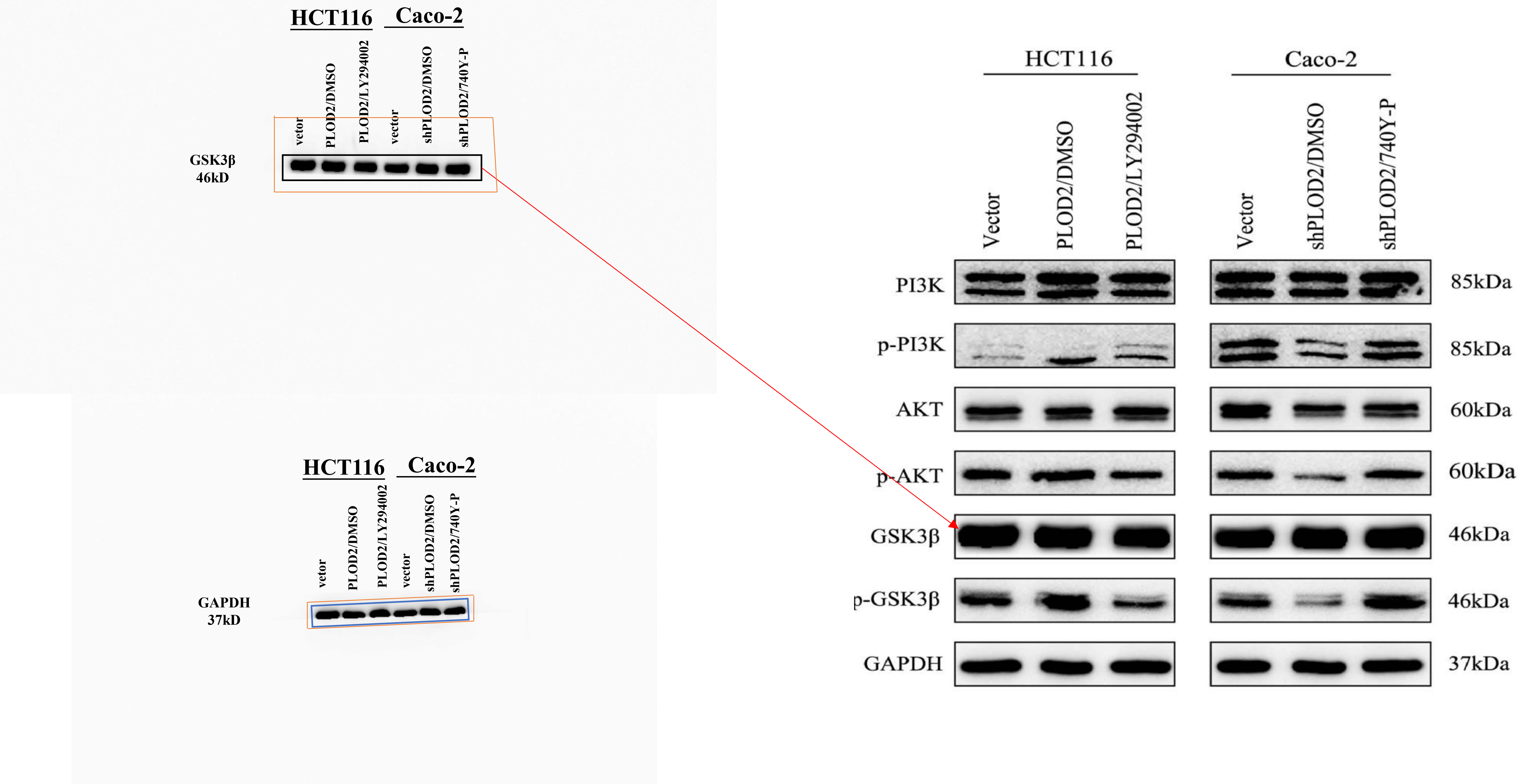
HCT116
Caco-2
vetor
PLOD2/DMSO
PLOD2/LY294002
vector
shPLOD2/DMSO
shPLOD2/740Y-P
GSK3β
46kD
HCT116
Caco-2
vetor
PLOD2/DMSO
PLOD2/LY294002
vector
shPLOD2/DMSO
shPLOD2/740Y-P
GAPDH
37kD
HCT116
Caco-2
Vector
PLOD2/DMSO
PLOD2/LY294002
Vector
shPLOD2/DMSO
shPLOD2/740Y-P
PI3K
p-PI3K
AKT
p-AKT
GSK3β
p-GSK3β
GAPDH
85kDa
85kDa
60kDa
60kDa
46kDa
46kDa
37kDa

Figure 4A

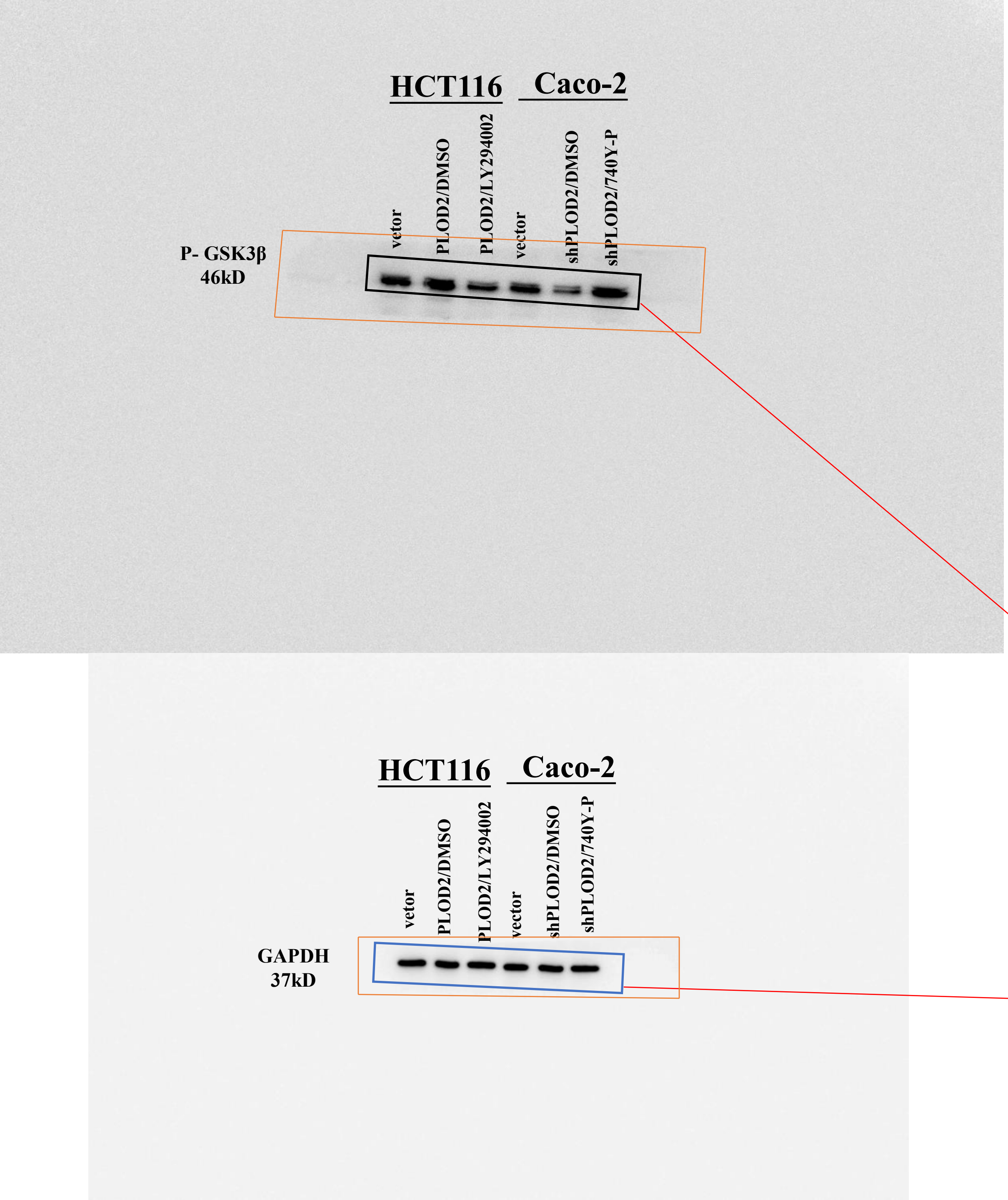
HCT116
Caco-2
vetor
PLOD2/DMSO
PLOD2/LY294002
vector
shPLOD2/DMSO
shPLOD2/740Y-P
P- GSK3β
46kD
HCT116
Caco-2
vetor
PLOD2/DMSO
PLOD2/LY294002
vector
shPLOD2/DMSO
shPLOD2/740Y-P
GAPDH
37kD

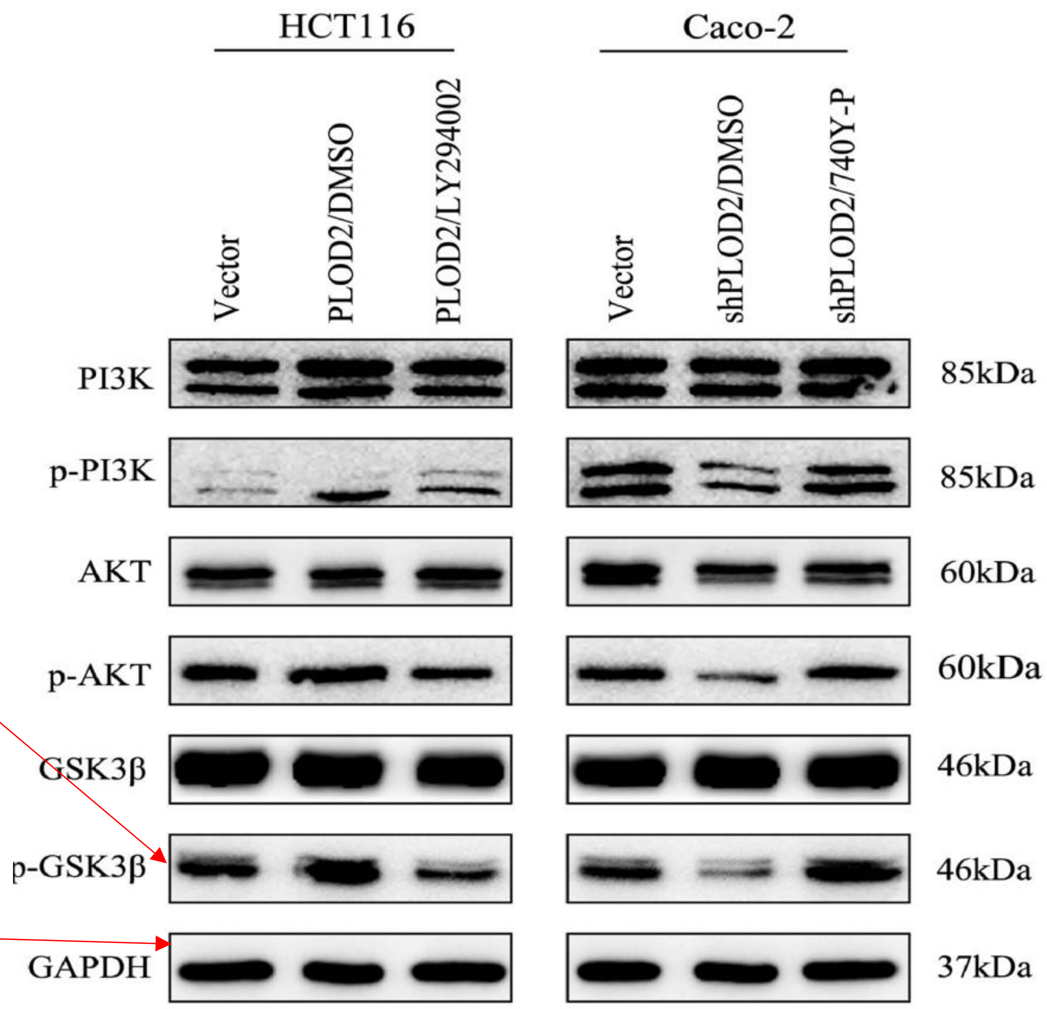
HCT116
Caco-2
Vector
PLOD2/DMSO
PLOD2/LY294002
Vector
shPLOD2/DMSO
shPLOD2/740Y-P
PI3K
85kDa
p-PI3K
85kDa
AKT
60kDa
p-AKT
60kDa
GSK3β
46kDa
p-GSK3β
46kDa
GAPDH
37kDa
